# Supplementary material for: Meta-analysis reveals an extreme “decline effect” in the impacts of ocean acidification on fish behavior
Source: PLoS Biol. 2022 Feb 3;20(2):e3001511. doi: 10.1371/journal.pbio.3001511 (PMC8812914; doi:10.1371/journal.pbio.3001511)
Supplement: S1 Table — Mean effect size magnitudes and their upper and lower confidence bounds for each dataset. Mean effect sizes were estimated by assuming a normal distribution and subsequently transforming the mean effect size using a folded normal distribution. Uncertainty around the mean effect size magnitude was estimated in a Bayesian fashion (see Materials and methods). mean mag = mean effect size magnitude; CI LB = lower confidence bound; CI UB = upper confidence bound. (DOCX) [file pbio.3001511.s015.docx]

**S1 Table. Mean effect size magnitudes and their uncertainty depicting the decline effect.**Mean effect size magnitudes and their upper and lower confidence bounds for each dataset. Mean effect sizes were estimated by assuming a normal distribution and subsequently transforming the mean effect size using a folded normal distribution. Uncertainty around the mean effect size magnitude was estimated in a Bayesian fashion (see Materials and methods). mean mag = mean effect size magnitude; CI LB = lower confidence bound; CI UB = upper confidence bound.

|  | Full dataset | | |  | Warm-water only | | |
| --- | --- | --- | --- | --- | --- | --- | --- |
| Year | mean mag | CI_LB | CI_UB |  | mean mag | CI_LB | CI_UB |
| 2009 | 5.4411 | 3.7220 | 7.3183 |  | 5.4400 | 3.6985 | 7.3527 |
| 2010 | 5.1959 | 4.2252 | 6.2583 |  | 5.1916 | 4.1935 | 6.2255 |
| 2011 | 0.3228 | 0.2261 | 0.4269 |  | 0.3237 | 0.2256 | 0.4282 |
| 2012 | 0.9008 | 0.7500 | 1.0578 |  | 0.9527 | 0.7859 | 1.1322 |
| 2013 | 1.0189 | 0.7215 | 1.3478 |  | 1.1591 | 0.8166 | 1.5442 |
| 2014 | 3.0191 | 2.5338 | 3.5185 |  | 3.0140 | 2.5480 | 3.5356 |
| 2015 | 0.2546 | 0.1941 | 0.3179 |  | 0.2409 | 0.1572 | 0.3364 |
| 2016 | 0.3916 | 0.3044 | 0.4825 |  | 0.4233 | 0.3254 | 0.5307 |
| 2017 | 0.3169 | 0.2515 | 0.3824 |  | 0.3849 | 0.2926 | 0.4834 |
| 2018 | 0.4425 | 0.3258 | 0.5764 |  | 0.2879 | 0.2433 | 0.3327 |
| 2019 | 0.0877 | 0.0540 | 0.1256 |  | 0.7364 | 0.0691 | 1.8973 |
|  |  |  |  |  |  |  |  |
|  | Olfactory cues only | | |  | Larvae only | | |
|  | mean mag | CI_LB | CI_UB |  | mean mag | CI_LB | CI_UB |
| 2009 | 5.4300 | 3.8001 | 7.3348 |  | 5.4300 | 3.8001 | 7.3348 |
| 2010 | 5.1881 | 4.2225 | 6.2426 |  | 5.1881 | 4.2225 | 6.2426 |
| 2011 | 0.3143 | 0.2104 | 0.4240 |  | 0.3143 | 0.2104 | 0.4240 |
| 2012 | 1.0780 | 0.8623 | 1.3074 |  | 1.0780 | 0.8623 | 1.3074 |
| 2013 | 1.0886 | 0.7544 | 1.4409 |  | 1.0886 | 0.7544 | 1.4409 |
| 2014 | 3.4758 | 2.8678 | 4.1220 |  | 3.4758 | 2.8678 | 4.1220 |
| 2015 | 0.2683 | 0.1957 | 0.3463 |  | 0.2683 | 0.1957 | 0.3463 |
| 2016 | 0.4139 | 0.3190 | 0.5203 |  | 0.4139 | 0.3190 | 0.5203 |
| 2017 | 0.3372 | 0.2589 | 0.4130 |  | 0.3372 | 0.2589 | 0.4130 |
| 2018 | 0.8193 | 0.5892 | 1.0552 |  | 0.8193 | 0.5892 | 1.0552 |
| 2019 | 0.0881 | 0.0519 | 0.1282 |  | 0.0881 | 0.0519 | 0.1282 |
