# Supplementary material for: Meta-analysis reveals an extreme “decline effect” in the impacts of ocean acidification on fish behavior
Source: PLoS Biol. 2022 Feb 3;20(2):e3001511. doi: 10.1371/journal.pbio.3001511 (PMC8812914; doi:10.1371/journal.pbio.3001511)
Supplement: S2 Table — Selected quotes pulled from 4 papers published in 2021 stating that ocean acidification is predicted to have broad impacts on fish behavior. (DOCX) [file pbio.3001511.s016.docx]

**S2 Table. Studies continue to reference early studies to state that ocean acidification is predicted to have wide ranging effects on fish behaviour and ecology.** Selected quotes pulled from four papers published in 2021 stating that ocean acidification is predicted to have broad impacts on fish behaviour.

| Quote | Reference |
| --- | --- |
| *“Elevated CO_2_ conditions can cause sensory deficits and altered behaviours in marine organisms, either directly by affecting end organ sensitivity or due to likely alterations in brain chemistry.”* | [1] |
|  |  |
| *“CO_2_-induced aquatic acidification is predicted to affect fish neuronal GABA_A_ receptors leading to widespread behavioural alterations.”* | [2] |
|  |  |
| *“For coral reef fishes, one of the most profound effects of ocean acidification is the impact on ecologically important behaviors.”* | [3] |
|  |  |
| *“Ocean acidification (OA), resulting from anthropogenic emissions of carbon dioxide (CO_2_), is predicted to impair sensory function and behaviour of fish.”* | [4] |
| [1] Radford et al. 2021, Proc. R. Soc. B., <https://doi.org/10.1098/rspb.2020.2754> | |
| [2] Hamilton et al. 2021, Sci. Tot. Environ., <https://doi.org/10.1016/j.scitotenv.2021.146320> | |
| [3] Vaughan & Dixson 2021, BioRxiv, <https://doi.org/10.1101/2021.01.23.427511> | |
| [4] Spatafora et al. 2021, Sci. Tot. Environ., <https://doi.org/10.1016/j.scitotenv.2021.149376> | |
