# Supplementary material for: Meta-analysis reveals an extreme “decline effect” in the impacts of ocean acidification on fish behavior
Source: PLoS Biol. 2022 Feb 3;20(2):e3001511. doi: 10.1371/journal.pbio.3001511 (PMC8812914; doi:10.1371/journal.pbio.3001511)
Supplement: S3 Table — Expanded details for each stage of the literature search, including results for each keyword and each database. Full search results can be accessed in S4 Data. (DOCX) [file pbio.3001511.s017.docx]

**S3 Table. Literature search results.** Expanded details for each stage of the literature search, including results for each keyword and each database. Full search results can be accessed in S4 Data.

|  | Search results | | |
| --- | --- | --- | --- |
|  | Google Scholar | Scopus | Combined |
| "ocean acidification fish behaviour" | 995 | 123 | 1118 |
| "ocean acidification fish behavior" | 993 | 123 | 1116 |
| "elevated co2 fish behaviour" | 998 | 90 | 1088 |
| "elevated co2 fish behavior" | 999 | 90 | 1089 |
|  |  |  |  |
| After duplicates removed | 2469 | 149 | 2508 |
|  |  |  |  |
| After initial screening for relevance | - | - | 93 |
|  |  |  |  |
| After detailed screening for inclusion | - | - | 88 |
|  |  |  |  |
| Added from cited reference search | - | - | 3 |
|  |  |  |  |
| Total articles included in meta-analysis | - | - | 91 |
