# Supplementary material for: Meta-analysis reveals an extreme “decline effect” in the impacts of ocean acidification on fish behavior
Source: PLoS Biol. 2022 Feb 3;20(2):e3001511. doi: 10.1371/journal.pbio.3001511 (PMC8812914; doi:10.1371/journal.pbio.3001511)
Supplement: Attachment — Submitted filename: Response to reviewers.docx [file pbio.3001511.s018.docx]

Dear Dr Clements,

Thank you very much for submitting your manuscript "An extreme decline effect in ocean acidification fish ecology" for consideration as a Meta-Research Article at PLOS Biology. Your manuscript has been evaluated by the PLOS Biology editors, an Academic Editor with relevant expertise, and by four independent reviewers.

You'll see that while some of the reviewers are broadly positive about your study, there are also some substantial concerns raised (especially by reviewers #1 and #4), and these must be addressed for further consideration. Broadly speaking, reviewers #1 and #3 work on marine ecology and fish behaviour, including the effects of acidification, and reviewers #2 and #4 are experts in meta-analysis (with an ecology background). Reviewer #1 is concerned about your treatment of two early outlier studies, both reviewer #2 and #4 ask that you use PRISMA reporting, and reviewer #4 has a number of other significant methodological concerns. Reviewer #3's requests are largely textual.

In light of the reviews (below), we will not be able to accept the current version of the manuscript, but we would welcome re-submission of a much-revised version that takes into account the reviewers' comments. We cannot make any decision about publication until we have seen the revised manuscript and your response to the reviewers' comments. Your revised manuscript is also likely to be sent for further evaluation by the reviewers.

We expect to receive your revised manuscript within 3 months.

**Response:** We thank the editors and the reviewers for the excellent feedback on our manuscript, and the chance to resubmit a revised version. We apologize for the delay in completing this revision; however, we wanted to ensure that all the detailed comments were carefully and properly considered. The comments have helped us improve all aspects of the manuscript, including the figures and analysis.

Generally, we have made substantial changes to the manuscript according to the reviewer comments. We also respond to each reviewer comment below in bold and provide line numbers to facilitate the second round of peer review.

Reviewer #1:

Summary: The current paper seeks to explore the possibility that ocean acidification research related to behavioral effects on fishes may suffer from a decline effect, and to also explore possible sources of this potential effect. They utilize a combination of qualitative and quantitative meta-analysis approaches to demonstrate (in their view) a decline effect, and to suggest that this effect is not due to biological processes. They conclude that ocean acidification does not have as a great an effect as previously thought. While I can see the rationale for this study, I honestly was not convinced by the work (for reasons I will outline) and was underwhelmed by their attempts to explain any observed effect, with the exception of the sample size discussion. There are a number of reasons, biological, methodological, and external, that may explain some of the observed shifts in effect size.

**Response:** We thank the reviewer for their detailed and critical review of our paper. The reviewer raises some great points regarding additional factors of interest that could potentially explain the decline effect. While we were unable to empirically test some of these factors, we have added an analysis that incorporates the potential role of life history stage (i.e., by analyzing data for larval experiments only). Furthermore, we have added an entire section of text that brings other potential moderators to light. The reviewer also provided very useful comments for tightening the language and writing, which we have incorporated as well.

Major Comments:

1. When looking at the data, it is clear that this entire trend is built on the presence of two early papers published on the topic, Munday et al 2010 and Dixson et al. 2010. I will obviously agree that the effect sizes in these papers are larger than typically found elsewhere in the literature, and this is not argued in the community. These papers were published a decade ago and to say that they are held up as representative of the field would be grossly inaccurate. But I have two other criticisms of the analysis with regard to these papers. First is that Dixson et al. 2010 appears to be classified under the 2009 data, which causes the 2009 reported value to be inflated in support of the decline effect. It might actually be included with both the 2009 and 2010 data. Confidence intervals are provided for both years, but one of the years should have only a single nested mean data point. A confidence interval can't be created around a single data. Anyway, I disagree with the use of the online date instead of official citation date (seriously, it was published online in late November). Dixson et al should be placed with the 2010 data, so the mean 2009 value would be 1.41 (the nested mean of Munday et al 2009, the only paper published that year). This all but destroys the decline effect trend as reported. This shows how sensitive the authors conclusions are to arbitrary choices made during data analysis.

**Response:** We thank the reviewer for these detailed comments. Firstly, the reviewer suggests that our statements regarding the influence of the early papers are misleading as they are no longer considered to be influential or representative of the field. A good indicator of influence within a given field is citations. We gathered Google Scholar citation metrics on September 10, 2021 for each of the 91 studies in the database and looked at two metrics of citations: citations per year, and citations since 2020 (the latter representing current citations). These data are presented in the figure below. The three studies in 2009-2010 are highlighted in grey. The red dashed line denotes the lowest citation metric for those three studies, and studies are ordered chronologically along the x-axis and colour-coded by year published online.

For both citation metrics (citations per year and citations since 2020), there are four studies that stand out as highly cited and hence highly influential, namely the initial three studies, together with Nilsson et al. 2012 (which also showed strong OA effects and proposed a neurological mechanism for those effects). While these studies may not be representative of results in current studies (which our decline effect suggests are small), it is abundantly clear that these early studies with very large effect sizes remain the most influential papers in this field today. We have revised the text in the manuscript throughout to reflect “influence” rather than perception of effects.

Furthermore, as the quotes below (all from papers published in 2021) show, researchers in this field are still approaching OA-fish behaviour studies through the lens of those early studies, with many directly stating that OA is “predicted” to have wide-ranging effects on behaviour. Coupled with the fact that the three early papers remain the most highly cited papers in this field, we strongly contend that the predictions in those early papers are still widely held by many (although not all) researchers in this field. At the very least, these predictions are still presented as widely held in recent publications. We have kept the text throughout to reflect this and have added a supplementary table of the following quotes for clarity:

- *“Elevated CO2 conditions can cause sensory deficits and altered behaviours in marine organisms, either directly by affecting end organ sensitivity or due to likely alterations in brain chemistry.”* (Radford et al. 2021, Proc. R. Soc. B., <https://doi.org/10.1098/rspb.2020.2754>)
- “*CO2-induced aquatic acidification is predicted to affect fish neuronal GABAA receptors leading to widespread behavioural alterations.*” (Hamilton et al. 2021, Sci. Tot. Environ., <https://doi.org/10.1016/j.scitotenv.2021.146320>)
- *“For coral reef fishes, one of the most profound effects of ocean acidification is the impact on ecologically important behaviors.”* (Vaughan & Dixson 2021, BioRxiv, <https://doi.org/10.1101/2021.01.23.427511>)
- *“Ocean acidification (OA), resulting from anthropogenic emissions of carbon dioxide (CO2), is predicted to impair sensory function and behaviour of fish.”* (Spatafora et al. 2021, Sci. Tot. Environ., <https://doi.org/10.1016/j.scitotenv.2021.149376>)

Next, we address the reviewer’s comments regarding online publication date. Online publication date represents the time at which scientists (and media) are first able to access a given piece of work. As such, this date represents the time at which a paper starts to influence a given field and public perception. Furthermore, the print date can often be far removed (> 1 year) from the date a paper was published online. We therefore maintain that the use of online publication date is more appropriate than using print date. In addition, using online publication date means that papers from online only journals and print journals are treated the same way in our analyses.

Nonetheless, we went ahead and re-analyzed the data by including Dixson et al. in the 2010 category, as suggested by the reviewer. Note that the error bars are now all above 0, as we have reanalyzed the data using a more appropriate technique as per comments from Reviewer 4 (given that this is a Bayesian approach using a folded normal distribution, statistical significance is not interpretable here).

The graph with Dixson et al. included in 2010 (i.e., using year printed rather than year online) can be viewed below:

As you can see in the figure, while this brings down the mean effect size for 2009, it does not “all but destroy” the decline effect, as the reviewer suggests. The decline effect is still very much evident regardless of what year we categorize the Dixson et al. paper.

We have thus elected to keep the year of online publication in the main graphs, as we feel that this best represents when a piece of work begins to influence a given field.

2. My second point about these two papers (this actually applies throughout) is the practice of nesting the effect sizes to generate a mean effect size per paper. Why is a mean valuable here? The data are clearly not normally distributed within a paper (nor is there any reason to believe they would be), so a mean is a poor representation of the combined data. This might be what the authors are referring to in the Figure 1 caption, but then they proceed to base their conclusions off of invalid overestimated means. If you calculate the median for these two papers they are 0.58 and 1.07, which are comparable to median values in later years. My argument is not to use the median, but in fact to use all the raw data. There is no need to reduce the data set with nesting, at least when attempting to initially identify a decline effect. When I generated a scatterplot of all the effect sizes by year, the decline trend is not apparent. There are effect sizes in excess of 13 as late as 2018. In other words, the identified decline effect is a product of selective data analysis by the authors, the trend is not robust to varying forms of analysis, and the trend is largely driven by two outlier papers published in the first two years.

**Response:** We appreciate the reviewer’s attention to varying forms of analysis. While the reviewer suggests that presenting mean effect sizes is inappropriate, this aligns with best practices and current analytical rigor in meta-analyses. The nested random assignment in the meta-analytical model simply accounts for the fact that individual observations are not independent of one another because measurements within a given study cannot be considered independent. Specifying this accounts for both within and between study variation when computing weighted mean effect sizes and their associated confidence intervals (which is indeed why you can still compute confidence bounds for a mean effect size when you have a single study for a given year). Furthermore, it is very important to understand that not all individual data points carry the same weight, as each data point needs to be weighted based on the sample size and variance of that given experiment. Moreover, it is not the independent observations that drive a study’s conclusions and interpretation. For example, while there were null effects detected in some of the early studies, the interpretation of those studies was that OA detrimentally affected behaviour. Instead, a study’s conclusions typically align with a weighting of the evidence from multiple experiments (as is the case in later studies from the same authors as the early studies, despite finding some significant effects). As such, regressing all individual data points over time may be useful for visualization, but is not a valid analytical approach here, and assessing study means is more appropriate.

Nonetheless, we have gone ahead and regressed individual data points over time, as suggested by the reviewer, to visualize whether or not this would negate the decline effect. The graph below depicts the absolute mean effect size for each individual experiment in our dataset, fit with a loess curve (now Fig 2a in MS):

As you can see, the decline effect is still very apparent in this regression of individual data points according to the loess curve. The general results also remain - very large effect sizes in 2009-2010, tapering to moderate effect sizes from 2011-2014, which eventually level out at small effect sizes after 2015. We thank the reviewer for this suggestion, and we now include the figure above in Fig. 2 of the manuscript for an additional visualization of the decline effect; but please note that there is no statistical analysis conducted on these data as this would be inappropriate. This trend remains unchanged if we regress mean effect size magnitudes for each study over time as well (data not included in the paper):

It is also important to note here that the means and confidence intervals computed for moderator levels in meta-analyses (i.e., year in our case) are weighted and do indeed incorporate all data points when computing weighted means and confidence bounds. As you will note, we have now re-analyzed the data with a more appropriate meta-analytical technique according to García-Roa et al. (2020), as per comments from Reviewer 4 (effect size magnitudes are tricky to deal with appropriately and require using a folded normal distribution and MCMC simulation procedures to properly estimate). The revised analysis uses what is known as the “transform-then-analyze” approach according to Morrissey (2016) and incorporates MCMCglmm to compute weighted mean effect size magnitudes and their confidence bounds. We have also consulted with a leading expert in this analysis, to confirm that the present analysis is indeed the best currently developed approach for this type of data.

We maintain that the data presented and the analyses used to compute and present effect sizes is the best approach. The identified decline effect is thus not a product of selective data analysis as suggested, but is based on valid and state-of-the-art meta-analytical approaches, and, as shown above, is very robust to multiple forms of analysis.

Finally, while we agree with the reviewer that a large part of the trend is driven by three early studies with high mean effect size magnitudes, mean effect sizes don’t seem to level off until 2015, with a notable bump in mean effect sizes from 2012-2014 as compared to 2015-2019. We thus maintain that the decline effect here is real.

**Reference:**
García‐Roa R et al. (2020) Biol. Rev. 95: 1607–1629.

3. The authors claim the observed trend (assuming it's true) cannot be explained biologically because they performed a cold water vs warm water comparison. The basis for this comparison is not explained, and it is not clear to me why cold water fish behavior would be less impacted by ocean acidification. This needs to be clearly articulated to the reader because to my understanding this comparison is not rooted in any sort of underlying physiology. Ocean acidification effects have nothing to do with metabolic rate.

**Response:** The main reason we investigated the effect of cold vs. warm water was that all early research was on tropical species. If cold water fishes proved highly tolerant and the proportion of cold-water species has increased over time, that could potentially have explained the decline effect detected. As the decline effect is present even when cold water fishes are removed from the dataset, that effect is insufficient to explain the decline effect.

Nonetheless, we apologize for not explaining our reasoning more clearly in the previous manuscript. In short, cold-water fish in temperate regions tend to experience a higher degree of temporal variability in carbonate chemistry parameters over large spatial areas (Kwiatkowski & Orr, 2018 *Nat. Clim. Change 8*, 141–145). Thus, increased variability in pCO2 in colder waters could result in a lower degree of sensitivity for cold-water species, generally speaking. If the decline effect were driven by increasing studies on more robust cold-water species (due to differences in temporal variation associated with latitude), the trend would disappear if those species were removed. While perhaps an imperfect proxy, studies in this field do not typically report on localized variability in seawater pCO2 conditions, and so there is no way of determining precise variability estimates for individual studies. Given that we restricted our data collection to pH changes applicable to IPCC projections, we feel that a comparison of cold- vs. warm-water species is valid. We have now clarified this in the text, along with the rationale for the other biological explanations (note that we have added life stage as a potential explanation as this reviewer suggested below):

**Lines 86-106:** *“The large effect size magnitudes from early studies on acidification and fish behaviour are not present in the majority of studies in the last five years (Fig. 1b; Table S1). This decline effect could be explained by a number of factors, including biological. For example, cold-water fish in temperate regions experience a higher degree of temporal variability in carbonate chemistry parameters over large spatial areas [15]. Therefore, they may be less sensitive to changes in seawater CO2 as per the Ocean Variability Hypothesis [16]. As such, if an increasing number of studies on cold-water species over time was responsible for the decline effect, removing cold-water species from the dataset (i.e., only including warm-water species) should result in the decline effect trend disappearing. This was not the case, as the decline effect persisted when only warm-water species were considered (Fig. 2a). In the same vein, the strongest ocean acidification effects on fish behaviour have undoubtedly been reported for chemical cue (herein ‘olfactory’) responses, and increasing numbers of studies on non-olfactory behaviours could explain the decline effect. If this was true, removing non-olfactory behaviours from the dataset should negate the decline effect trend. Again, this was not the case (Fig. 2b). Finally, early studies of ocean acidification and fish behaviour used larval fish, which are typically considered to be more sensitive to environmental perturbations than juveniles and adults. If a greater proportion of studies used less sensitive life stages through time, then removing those life stages and focusing exclusively on larvae should abolish the decline effect. Once again, this was not the case (Fig. 2c). These analyses show that ocean acidification studies on fish behaviour exhibit a decline effect that is not explainable by three biological processes commonly considered important drivers of acidification effects (Fig. 2a-c; Table S1).”*

4. Building on comment #2, Baumann 2019 (CJZ) has proposed that coastal species that experience high CO2 at various points in their life cycle are more resilient to its effects, and there is support for this line of thinking (i.e. acclimation and transgenerational plasticity). This seems like a much better biological test than a spurious latitudinal comparison. Furthermore, the authors must also address the increased use of captive bred fish populations. High CO2 is a very well-known problem in aquaculture, and fish reared in captivity could be acclimated to such conditions, or even selected to perform well in such conditions.

**Response:** While we agree with the reviewer that there are many other potential explanations for the decline effect, these are not readily testable based on available information in papers to date. For example, explicitly testing for natural pCO2 variability is not feasible because background pCO2 variability is not available for the vast majority of studies. However, studies show that pCO2 variability increases with increasing latitude, which is why we included the temperate/polar (cold water) vs. tropical/subtropical (warm water) comparison.

While we couldn’t explicitly test these ideas empirically as suggested, we have added a section to the paper that details other potential explanations for the observed decline effect that require more data to empirically test. We have worked these into a call for more comprehensive methodology and data collection:

**Lines 113-127:** “*While we were able to test and exclude three biological factors, there are other potential factors that could drive the decline which are not readily testable from our database. For example, while we were able to partially test for the influence of background CO2 variability by comparing cold- and warm-water species, most studies do not report the actual background CO2 levels that the experimental animals (and their ancestors) have historically experienced. As such, we are unable to account for the historic CO2 acclimation conditions of animals used in experiments. The impact of this with respect to the observed decline effect could stem from an increasing proportion of studies using captive bred fish from recirculating aquarium systems with high CO2 levels, as compared to fish from wild populations experiencing natural CO2 levels. This is an unlikely explanation for the decline effect, however, given that the earliest studies conducted in 2009–2010 reporting high effect sizes were conducted with both captive-bred and wild-caught fish [7–9, 13]. Furthermore, recent replication attempts of those initial studies using wild-caught fish have failed to replicate the large effect sizes [6]. Nonetheless, we recommend that future studies provide better background CO2 information for the fish used in their experiments, and use best practices for measuring and reporting carbonate chemistry [15].*”

5. The authors propose that selective publication bias may be, in part, to blame for the decline effect. I would argue the exact opposite. Ocean acidification is unique in environmental science as a topic in which you can readily publish a completely negative results data set (in Nature no less). I would challenge the authors to find any other environmental stressor where this publication access is available to scientists. I am not arguing it is a bad thing, negative results are valuable in the context of climate change, but the authors ignore the fact that the field has been littered with low impact journal no-effect papers. In fact, the authors cite a paper by Browman (2016 ICES Marine Science) that served as a "call-to-arms" to publish negative results papers and ICES is an excellent place to do it. So my question is this: is the observed decline effect because of an atypical decrease in effect size, or is it because of an atypical number of no-effect publications that would not have been publishable under different circumstances. For example, is a no-effect CO2 paper on fish behavior publishable in 2006? Probably not. Again, I would argue the authors are not viewing their data set impartially. The field seems to have settled in a good place, behavior effects are not pervasive but may be a part of the overall ocean acidification story. Isn't this the way the system is supposed to work?

**Response:** We generally agree with the reviewer’s reasoning, and perhaps we did not convey some of these points clearly in our original manuscript. Based on the spread of data in the new scatterplots that we provide, we suggest that the decline effect is driven likely by both of the points raised by the reviewer. Looking at the scatterplots generally, there are fewer studies with small weighted mean effect sizes and more studies with larger effect sizes (>1) prior to 2015, after which there were comparatively less studies with large effect sizes and comparatively more studies with lower effect sizes. Indeed, 12 studies had weighted mean effect sizes >1 prior to 2014, while only three studies had weighted mean effect sizes >1 subsequently. Indeed, the two special issues in ICES were published almost precisely when effect sizes for this field leveled out – the special issues promoted null and negative effects because many had recognized that the field likely suffered from publication bias. In fact, the solicitation email titled the special issue “Towards a broader perspective on ocean acidification research – jumping off the bandwagon” (<https://news-oceanacidification-icc.org/2014/06/16/ices-journal-of-marine-science-invitation-to-contribute-towards-a-broader-perspective-on-ocean-acidification-research/>). This suggests that the decline effect is likely driven by a combination of fewer studies reporting large effect sizes in later years (and more studies reporting small effect sizes), and early studies publishing large effect sizes.

We agree with the reviewer that the field has likely settled in a good place, which is positive. However, studies continue to cite the early studies on acidification and fish behaviour more than others, often using them to promote the idea that acidification will have wide-ranging effects on fish behaviour and ecology (i.e., see quotes in specific comment 1 below, and in Table S1). We have emphasized this point further in the revised version with an added statement in the “Final Remarks” section:

**Lines 266-270:** “*While the field has seemingly settled in a good place with respect to realistic effect sizes, it has taken 10 years to get there. Furthermore, studies continue to cite early studies with unreasonable effect sizes to promote that acidification is predicted to broadly impact fish behaviour and ecology (e.g., Table S2), suggesting that a shift in mindset is still needed for many in this field.*”

The question remains, however, how was it possible for early studies to report extremely large effect sizes if such large effects don’t actually exist? We make the point that it has taken 10+ years and a lot of money to settle in the “good place”, and there are steps that could be taken to reduce the amount of time and money required to reach a more realistic empirical truth.

6. I see no benefit to the qualitative analysis. You have quantitative data to rely on.

**Response:** This was also suggested by another reviewer. We have thus removed the qualitative analysis as suggested.

7. I would have liked to see some attempt to divide the data based on behavior type. The authors did try to address this a little bit with the baseline vs cue data, but I would argue that it really isn't mechanistically valid to combine olfactory endpoints, lateralization endpoints and anxiety type endpoints in a single analysis. Or at the very least, they should also be analyzed separately.

**Response:** This is a valid point. Undoubtedly, the strongest OA effects have been observed for olfactory responses, and this type of response is certainly the most studied. As such, if the decline effect was driven by an increasing number of studies measuring non-olfactory responses, the observed decline would not be apparent if we only included olfactory responses. We have re-analyzed the data accordingly. Rather than using “stimulus vs. no stimulus” as we did in the previous version of this manuscript, we reanalyzed the data using only experiments with olfactory cues. This included experiments assessing responses to predators, prey or other food, olfactory habitat cues, and kin (including mates, conspecifics, and offspring). This analysis yielded the same general results as the previous analysis, as depicted in the graph below (Fig 3b in revised MS):

8. This wasn't mentioned by the authors, but I've always wondered whether the shift in sensitivity had anything to do with differences in the way CO2 was calculated. Munday et al 2010 used a submerged gas probe with a permeable membrane, as opposed to the many studies that quantify pH and titratable alkalinity. Dixson only relied on pH measurements (no alkalinity) and doesn't delineate what type of pH scale it is. The real PCO2 could be off by quite a bit from what is reported. That method would never be published today. If the authors are actually interested in looking at all possible explanations for this outlier paper (and I think their decline effect is more an outlier paper effect), I think it is reasonable to include the analytical chemistry as an explanation.

**Response:** We agree with the reviewer that the real pCO2 can be quite different from what is reported when only pH has been measured. In fact, it has been shown that measurements made with a pH probe can be as far off as 0.1 pH units even when measured in state-of-the art laboratories (Bockmon and Dickson 2015). This is definitely a point of concern in any paper that does not perform direct measurements of pCO2. However, even if the real pCO2 can be quite different to the reported values, there would still be a true difference in pCO2 between the control group and the high CO2 group (although papers testing intermediate levels may well have overlapping CO2 treatment groups). Since the errors in pCO2 due to different measuring techniques would maybe equate to ~300 μatm variability (real vs. estimated), it is highly unlikely that such a discrepancy could be responsible for the observed decline effect. Furthermore, two of the three initial papers in this field (Munday et al. 2009, 2010) used precise pCO2 measurements (Vaisala pCO2 probes).

Ultimately, we feel that this is a very unlikely cause of the decline effect, and elaborating on this in the manuscript would be tangential. We have thus elected not to expand on this in the MS.

**Reference:**
Bockmon et al. (2015) Mar. Chem. 171: 36-43.

9. Throughout the manuscript the authors place a major emphasis on the effect size, presumably because greater effect sizes are related to more detrimental impacts of ocean acidification. But the authors should be careful to not equate these things too directly. For example, just say instead of impacting 100% of individuals, OA will only impact 30%. Is that ecologically meaningless? In 2018 the effect size 95% CI does not overlap with zero, so there is still an effect there. The ecological significance of the effect is the question.

**Response:** It is not that larger effect sizes necessarily mean more detrimental impacts - we want to be clear that behavioural responses are complex and there are often multiple functional ramifications of a given behaviour. We have now made this explicit in the text and revised the text thoroughly to avoid relaying a sentiment of “detrimental” or “outstanding” to tone down the language. Nonetheless, larger effect size magnitudes do indeed represent stronger behavioural effects of OA, which are often interpreted as consequential for ecology (as some of the quotes provided below in specific comment 1 show).

Regarding the reviewer’s statement “...just say instead of impacting 100% of individuals, OA will only impact 30%.”, the main difference is that 100% impact implies zero acclimation/adaptation capacity, whereas 30% impact would mean there is variability/plasticity within the population. This variability could imply that organisms have the ability to phenotypically/genotypically adjust and adapt to the change in pCO2. Nonetheless, we have removed the qualitative analysis that refers to “% of studies with X effect”.

Finally, given the re-analysis as per comments from Reviewer 4, we have removed all mention of “statistical significance”, as interpreting significance based on CI bounds overlapping with 0 is no longer valid.

Specific Comments:

1. Line 16: "is expected to". This wording is misleading of the field. Ocean acidification has been intensely studied and its effects are varied. I would argue that the general community views ocean acidification as much less of a threat than a decade ago, but that there are specific circumstances where it may cause problems.

**Response:** The text has been revised:

**Lines 16-17:** “*Ocean acidification – decreasing oceanic pH resulting from the uptake of excess atmospheric CO_2_ – has the potential to affect marine”*

However, as the quotes below (all from papers published in 2021; now contained in Table S2) show, researchers in this field are still approaching OA-fish behaviour studies through the lens of those early studies, with many directly stating that OA is “predicted” to have wide-ranging effects on behaviour. Coupled with the fact that the three early papers remain the most highly cited papers in this field, we strongly contend that the predictions in those early papers are still widely held by many (although not all) researchers in this field. At the very least, these predictions are still presented as widely held in recent publications. We have kept the text throughout to reflect this and have added a supplementary table of the following quotes for clarity:

- *“Elevated CO2 conditions can cause sensory deficits and altered behaviours in marine organisms, either directly by affecting end organ sensitivity or due to likely alterations in brain chemistry.”* (Radford et al. 2021, Proc. R. Soc. B., <https://doi.org/10.1098/rspb.2020.2754>)
- “*CO2-induced aquatic acidification is predicted to affect fish neuronal GABAA receptors leading to widespread behavioural alterations.*” (Hamilton et al. 2021, Sci. Tot. Environ., <https://doi.org/10.1016/j.scitotenv.2021.146320>)
- *“For coral reef fishes, one of the most profound effects of ocean acidification is the impact on ecologically important behaviors.”* (Vaughan & Dixson 2021, BioRxiv, <https://doi.org/10.1101/2021.01.23.427511>)
- *“Ocean acidification (OA), resulting from anthropogenic emissions of carbon dioxide (CO2), is predicted to impair sensory function and behaviour of fish.”* (Spatafora et al. 2021, Sci. Tot. Environ., <https://doi.org/10.1016/j.scitotenv.2021.149376>)

2. Line 18: "most catastrophic". Again, this is a misrepresentation. The people that study behavior (like the authors) place it amongst the most catastrophic effects, but I'm not sure the entire community agrees. I would suggest to tone down the language here.

**Response:** The text has been revised to tone down the language, but we again maintain that may researchers in this field continue to approach their studies through the lens of broad ranging behavioural effects of OA (see example quotes in the comment above from papers published in 2021). The sentence now reads as follows:

**Lines 17-19:** “*Among the possible consequences, a series of studies on coral reef fishes suggested that the direct effects of acidification on fish behaviour may be extreme and have broad ecological ramifications.*”

3. Line 19: "dire prediction". The authors are framing this manuscript as if it is 2011. These dire predictions are not roundly held by the community. There are a number of review papers that have challenged the GABA hypothesis, and pointed out that the effects of OA on behavior are not widely observed amongst fishes, at least at OA relevant CO2 levels.

**Response:** We have removed the word “dire” from this sentence to tone down the language again, we maintain however that this is a prediction that remains prevalent in the field (see example quotes in the comment above from papers published in 2021). The sentence now reads as follows:

**Lines 19-21:** “*Recent studies documenting a lack of effect of experimental ocean acidification on fish behaviour, however, call this prediction into question.”*

4. Line 23: You tried one thing. This is a gross overstatement.

**Response:** The text has been revised for precision:

**Lines 21-23:** “*Here, we explore the consistency and robustness of scientific evidence over the past decade regarding direct effects of ocean acidification on fish behaviour by testing for a “decline effect”.”*

5. Line 25: This conclusion is again rooted in 2011. There is unanimous understanding that the effects of OA on behavior are not universal, and as such the early "dire" predictions were abandoned year ago. That isn't to say that there are not behavior based effects that can have serious consequences. I would tone this down to be more representative of the field.

**Response:** Based on the quotes from papers published in 2021 in our response above (Table S2 in MS), and the fact that the three early studies remain the most highly cited in this field, even when only counting citations since 2020, we respectfully disagree with the reviewer that current research in this field has abandoned widespread dire predictions of OA on fish behaviour. We have thus elected to keep the text as is here:

**Lines 30-32:** *“We contend that ocean acidification has a negligible direct impact on fish behaviour, and we advocate for improved approaches to minimize the potential for a decline effect in future avenues of research.”*

6. Line 29: I was a little surprised that a paper on OA effects didn't really introduce the topic in a meaningful way.

**Response:** The concept of ocean acidification, at this point in time, is well known by most scientists. We generally describe what ocean acidification is in the abstract and feel that this brief description should suffice. Given the generality of this concept, we do not feel that reiterating the specifics regarding OA projects, etc. is useful. We are willing to provide such background information if the reviewer and editor feel it is necessary but have elected to keep the focus on OA-fish behaviour content.

7. Line 37: Rephrase. Inflated initial reports infers some sort of deliberate intent, which I don't think is what the authors are trying to say. Also applies to the next sentence.

**Response:** The text has been amended in both sentences:

**Lines 42-44:** *“It captures the concept of initial reports with large effect sizes that overestimate reality. In such instances, the early, large effect sizes are the key problem, not the subsequent decline. The decline effect could therefore equally be referred to as the ‘early inflation effect’.”*

8. Line 47: Again, this subheading infers deliberate intent.

**Response:** The subheading has been removed in accordance with the reviewer’s suggestion.

9. Line 48: I would argue that very few have documented negative effects. That requires a very high power, which most studies do not have. The authors cannot argue for the importance of sample size for type 1 error but ignore it for type 2 error. In reality, most of the no effects papers are unable to demonstrate a positive effect but inconclusive with regard to no effect.

**Response:** In this sentence, “negative effects” referred to functionally-negative impacts of OA on biological processes, not a failure to detect an effect of OA. We have revised the text for clarity:

**Lines 47-48:** *“Over the past 15 years, biologists have documented substantial impacts of ocean acidification on marine biota [5].”*

10. Line 50: I would say temperature is a pretty nice precedent. Or maybe the effects of oil following the Exxon oil spill, or Deepwater Horizon. Maybe the current explosion of PFAS work. I know PLOS Biology is a high impact journal, but the hyperbole in this manuscript is over the top.

**Response:** The reason we cite the Browman paper here is because that paper states that the growth of the OA field is “unprecedented in marine science”. We have revised the text here, but want to stress that we are using previously-supported claims and not over-the-top hyperbole as the reviewer suggests:

**Lines 48-50:** *“With more than 300 papers published per year from 2006 to 2015, the exponential growth of ocean acidification studies represents one of the fastest expanding topics in the marine sciences [6], and underscores the perceived risk of ocean acidification to ecosystem resilience.”*

11. Line 54: outstanding to large?

**Response:** We’ve revised the text accordingly:

**Lines 53-55:** *“Some of the most striking effects of ocean acidification are those concerning fish behaviour, whereby a series of sentinel papers in 2009 and 2010 published in prestigious journals reported large effects of laboratory-simulated ocean acidification [8–10].”*

12. Line 62: I'm pretty sure the universality of behavioral effects was abandoned long ago.

**Response:** As we have detailed in our responses above, studies still in 2021 reference and state that OA is predicted to have wide ranging behavioural effects. We have largely kept the text as is here, however removing reference to “universality”, following the suggestion by the reviewer:

**Lines 62-64:** *“In recent years, however, an increasing number of papers have reported a lack of ocean acidification effects on fish behaviour, calling into question the reliability of initial reports.”*

13. Line 65: This might be a good place to actually expand on the types of behaviors.

**Response:** We have added a few words to state that studies expanded into a range of behaviours, details about types of behaviours is described:

**Lines 59-62:** *Not only were the findings alarming, the extraordinarily clear and strong results left little doubt that the effects were real, and a multimillion-dollar international investment of research funding was initiated to quantify the broader impacts of ocean acidification on a range of behaviours.”*

14. Line 99: How is an increased study of cold water species a likely culprit? This makes no sense whatsoever.

**Response:** This has been addressed as per the response to Major Comment 3 above.

15. Line 177: Again, negative results is a misnomer. Most studies in biology do not have the sample sizes to truly say if something is a negative result. That's why p-value is more highly emphasized then power after running the statistical tests (not to be confused with power analysis during experimental design).

**Response:** In accordance with the suggestion by the reviewer, we have amended the text the term “negative effect”:

**Lines 237-238:** *“First and foremost, we suggest that journals adopt the practice of registered reports to ensure that studies not detecting an effect are published in a timely manner.”*

16. Line 214: Why would you exclude freshwater species? The field is concerned with the effects of CO2 on behavior. Salinity has no bearing on how blood chemistry is altered, nor how the olfactory epithelium functions. This begs the question of how the "decline effect" results would hold up with the two excellent paper showing olfactory and anxiety in salmon, or the reverse OA effects observed in catfish (both high impact studies). Those were published in 2015 and 2016. At the very least an analysis that includes them should also be included for the readers to evaluate.

**Response:** Our paper concerns ocean acidification, and the chemistry of freshwater and seawater are so different that the two systems and processes are not comparable. Furthermore, there are indeed differences in physiology between freshwater and marine species that do not allow for direct comparisons (Ishimatsu et al. 2008). Additionally, the change in pH in freshwater acidification studies most often far exceeds the change in pCO2 in marine environments. Adding the two excellent studies (out of hundreds of studies on freshwater species and acidification) that the reviewer suggests would be a very biased approach, and we have hence not included them. We have thus elected to keep our dataset focused on the marine realm.

**Reference:**

Ishimatsu A et al. (2008) Mar. Ecol. Prog. Ser. 373: 295-302.

17. Line 231: I wish something was done with the life stage data. One potential biological issue here is a shift away from sensitive life stages to more resilient ones. It is well established that early life stages are more sensitive to a number of environmental issues because the increased surface area to volume ratio.

**Response:** This is a good suggestion. As the reviewer suggests, larvae might be more sensitive to OA than juveniles and adults (however, several papers on adult fish and OA effects do not support that suggestion, e.g., Cripps et al 2011, Devine et al. 2012). If the decline effect would be driven by increasing studies on more tolerant life stages, the trend should disappear if we only include larvae in the analysis. Indeed, in the revised version of the manuscript we have included such an analysis in the paper. The graph for the larvae-only dataset can be viewed below:

As you can see, the decline effect is still strongly evident when only larvae are included. Furthermore, the strong increase in effect size in 2014 in the other datasets disappears when we only include larvae, indicating that the increased effect size for 2014 cannot be explained by sensitive larvae (i.e., the increased response in 2014 is driven by effects on more tolerant life stages). This provides further evidence that the decline effect is not explainable with biological factors such as life stage.

We now include this analysis in the revised manuscript and written results:

**Lines 99-103:** *“Finally, early studies of ocean acidification and fish behaviour used larval fish, which are typically considered to be more sensitive to environmental perturbations than juveniles and adults. If a greater proportion of studies used less sensitive life stages through time, then removing those life stages and focusing exclusively on larvae should abolish the decline effect. Once again, this was not the case (Fig. 2c).”*

**References:**

Cripps IL et al. (2011) PLOS One 6: e22736

Devine BM et al. (2012) Oecologia 168: 269-276

18. Line 320: Should probably be more specific regarding the variance cut off threshold.

**Response:** This aspect of the paper has been removed as we have re-analyzed the data with a more robust method (in accordance with comments from Reviewer 4), which computes effect size variance in a different way.

19. Line 332: "and if temperate species are tolerant". But why would the authors have this expectation?

**Response:** This has been addressed as per the response to Major Comment 3 above.

Reviewer #2:

I really enjoyed reading this paper. It is clear and very accessible. The research question is also highly current and relevant. The paper make an excellent case study, and I could easily see myself using it in my lectures on research ethics. Below I make some minor comments as well as some suggestion for additional analyses.

**Response:** We thank the reviewer for their positive assessment of our manuscript and detailed comments for improvement.

1. Line 15: typo - should be "decreasing"

**Response:** Nice catch! Typo has been fixed:

**Lines 16-17:** “*Ocean acidification – decreasing oceanic pH resulting from the uptake of excess atmospheric CO2 – has the potential to affect marine life in the future.*”

2. Line 130 - 131: I agree that bias can skew results, but you don't actually have any evidence to show biased behaviour in your study. Whether patterns are due to confirmation bias would be very difficult to demonstrate. Given this I think you need to think about whether and how you make this point, to make sure it doesn't imply you have evidence showing that experimenter bias was at play. Having said that it might be possible (and would be interesting/relatively easy) to look at whether there is any evidence that effect sizes are greater when studies were conducted blind or not…

**Response:** This is a valid point. While we don’t have empirical evidence for this from our analysis, we do feel that it is an important potential source of error to include. We have added text to explicitly state that we do not demonstrate experimenter bias empirically, but that none of the three initial studies with large effect sizes contained a statement of blinding. In fact, few studies do include a statement of blinding, and if such is provided, no details are given to how blinding was achieved (which is particularly important in OA research given that the CO2 dosing system is both auditory and visually evident to the observer). Overall, the text is now more cautious regarding this point:

**Lines 160-171:** *“Experimenter/observation bias during data collection is known to seriously skew results in behavioural research [16]. For example, non-blinded observations are uncommon in life sciences, but are known to result in higher reported effect sizes and more significant p-values than blinded observations [17]. Most publications assessing ocean acidification effects on fish behaviour, including the initial three studies reporting large effect sizes, do not include statements of blinding for behavioural observations. Furthermore, given that statements of blinding can be misleading [18], there has been a call for video evidence in animal behaviour research [19]. Moreover, the persistence of inflated effects beyond initial studies can be perpetuated by confirmation bias, as follow-up studies attempt to confirm initial inflated effects and capitalise on the receptivity of high-profile journals to new (apparent) phenomena [20]. While our analysis does not empirically demonstrate that experimenter bias contributed to the decline effect, it is possible that conscious and unconscious experimenter biases may have contributed to large effect sizes in this field.”*

3. Line 142 - 147: I'm not sure I get the purpose of this analysis or that I quite understand what you are trying to get at (see also comment in methods section). I feel like the journal impact factor stuff is also kind of conflated with time since publishing a novel result… I wonder if it would be better to use funnel plots to look for publication bias. To me it feels like what's going on is just general publication bias (i.e. studies with small sample size only get published if there is a significant result and not if it is null) and doesn't necessarily need to invoke journal impact factor (apart from high impact studies being more likely to publish novel results). The fact that the journal impact factor has decreased over time is probably true for any novel result/anything that's published in a high impact journal. I guess I'm just not sure it tells you anything interesting.

**Response:** Thanks to the reviewer for their comments here. We can’t really use funnel plots to detect publication bias due to using effect size magnitudes. However, we have restricted our analysis for “bias” to highlight exactly what the reviewer states: studies with the largest effect sizes tend to have low sample size, but are published in high IF journals and have fuelled citation bias in this field. While the reviewer qualifies this as ‘general publication bias”, we do feel that this is important to highlight quantitatively. Nonetheless, we agree with the reviewer that looking at trends in impact factor and sample size over time does not provide anything more substantial and have removed this from the manuscript.

4. Line 150: typo - "fuel"

**Response:** Correction made:

**Lines 179-180:** “*This biased publishing can result in the proliferation of studies reporting strong effects, even though they may not be true [21], and can fuel citation bias [22].*”

5. Line 151: I think this citation bias that might be published by stuff published in high journals is potentially very interesting, and perhaps could be looked into further. It might be quite nice to follow this idea through. Line 153 - I don't see how you sample size analysis tells you anything about how much these papers have been cited… do you mean because high impact journals are cited more? But that doesn't necessarily mean these specific studies are cited more. That is very easy data to get though. Perhaps you could compare citations for papers from different impact factors while controlling for Year or something?

**Response:** We agree with the reviewer that the number of citations per paper could be more thoroughly investigated (this was also requested by Reviewer 1). We have hence gathered Google Scholar citation metrics (as of September 10, 2021) for each of the 91 studies in the database and looked at two metrics of citations: citations per year, and citations since 2020 (the latter representing current citations).

The text has been thoroughly revised and we have focused our attention here on actually showing citation bias. Note that the studies with the highest citations per year, and highest citations since 2020, include the initial 3 studies in this field and one other study in 2012 (see panels a-b in figure below; this is now Fig. 4 in the manuscript). In addition, we show that larger effect sizes in this field are generally published in higher impact journals, and that those studies are cited more than null effect studies in lower impact journals (panels c-d in figure below):

The text surrounding publication and citation bias new reads:

**Lines 180-193:** *“To determine if studies testing for effects of ocean acidification on fish behaviour exhibited signs of publication bias and citation bias, we assessed relationships between effect size magnitude, journal impact factor, and Google Scholar citations (Fig. 4). Examining average citations per year and the total number of citations since 2020, four papers stood above the rest: the initial three studies in this field [8–10] and the sentinel paper proposing GABA¬A neurotransmitter interference as the physiological mechanism for observed behavioural effects [27] (Fig. 4a,b). While it is difficult to quantify whether authors selectively published only their strongest effects early in this field, we were able to quantify effect size magnitudes as a function of journal impact factor. We found that the most striking effects of ocean acidification on fish behaviour have been published in journals with high impact factors (Fig. 4c). In addition, these studies have had a stronger influence (i.e., higher citation frequency) on this field to date than lower impact studies with weaker effect sizes (Fig. 4b,c). Similar results have been reported in other areas of ecology and evolution, perhaps most notably in studies regarding terrestrial plant responses to high CO2 [28].”*

6. Line 165 & 166: I don't think you show any evidence that supports the claim of observer bias. This early pattern could be solely driven by low sample sizes in combination with publication bias

**Response:** The reviewer is correct. We have amended the text in this section accordingly:

**Lines 214-223:** *“Our results suggest that large effects of ocean acidification on fish behaviour were at least in part due to methodological factors in early studies (e.g., low sample sizes). Furthermore, the proliferation and persistence of this idea has likely been aided by the selective publication of large effect sizes by authors and journals, particularly at the onset of this field, and the continued high frequency of citations for those papers. It is important to note, however, that low sample size and selective publication cannot fully explain the strong decline effect detected here, and other biases and processes may be at play [7,29]. Nonetheless, we call on journals, journal editors, peer-reviewers, and researchers to take steps to proactively address the issues of low sample size and selective publication, not only in the ocean acidification field, but also more broadly across scientific disciplines.”*

7. Line 170: this is a great recommendation, but it is made in hindsight, knowing what is a good sample size... is it possible to make a more general minimum sample size recommendation? Even 30 feels on the low side for a new finding that has never been shown before. Or a call to increase sample sizes more quickly once first study is out, so that the literature is rectified earlier? I was surprised that it took 5 years to start seeing decent sample sizes in the literature... if science is working shouldn't the first thing after seeing a novel result in the literature be to see if the result is robust... Well ideally people might think to do that before publishing a novel result for the first time... but if not then.

**Response:** Unfortunately, sample size recommendations are commonly made in hindsight given that adequate sample sizes and realistic effect sizes are not known *a priori*. We certainly agree with the reviewer that this is a serious issue. Performing pilot trials to enable power analyses to be conducted might be one way forward, and we now suggest this in the text (see text below). While we agree with the reviewer that 30 might be on the low side, but this is the sample size threshold after which extreme effects are not commonly observed:

Nonetheless, we have incorporated the reviewer’s nice suggestion to call for studies with larger sample sizes earlier into the revised text:

**Lines 229-235:** *“In addition, for other fields, we suggest that studies with higher sample sizes be published alongside, if not very soon after, an original novel finding to ensure that such a finding is robust. Ideally, researchers would conduct pilot studies with varying sample sizes to determine an adequate sample size threshold; however, time and financial constraints can make this difficult. While adequate sample sizes will vary across topics and fields, ensuring studies with large sample sizes are published early alongside those with smaller sample sizes can strive toward reducing the amount of time it takes to truly understand a phenomenon.”*

8. Line 177: I'm not really sure how pre-registration ensures all negative results are published in a timely manner. I remember reading (sorry can't remember where) that pre-registered studies often go unpublished. I do see that pre accepting papers based on their methods rather than there results could help with publication bias though.

**Response:** Thanks for this comment! Indeed, we were thinking along the lines of journals pre-accepting articles based on their methods. We have added clarity to the text here and have specified “registered reports” rather than pre-registration:

**Lines 237-244:** *“First and foremost, we suggest that journals adopt the practice of registered reports to ensure that studies not detecting an effect are published in a timely manner. Herein, journals should provide authors with the ability to submit proposed methodologies and have them formally peer reviewed prior to studies even being conducted. If methodologies are deemed sound (or revised to be so) and “accepted” by reviewers, journals should commit to publishing the results regardless of their outcome. While not a silver-bullet solution, this practice could help to minimize selective publication bias and reduce the risk of early, flawed studies being disproportionately influential in a given field [30].”*

9. Line 199: I think that it would be worth being more explicit about what the overall effect size is for your data... Seems to me that for the set of traits you look at there is probably no effect, or at most a very weak effect. You could have a decline effect when the effect of interest is still important and I think it is worth reporting the average effect size for your study, as well as perhaps doing some analyses that demonstrate how much of an influence this effect can have on our overall findings and for how long

**Response:** We appreciate this suggestion; however, given the very large effect sizes detected in the early studies, we feel that an overall effect size magnitude for the field would still be overestimated and comparing annual effect sizes to this number would be misleading. Additionally, because we provide evidence that bias has likely influenced results in this field, an overall mean effect size would be inaccurate. We have thus elected to refrain from including this analysis and retain comparing the effect sizes of early studies to the more realistic effect sizes observed over the past five years (Fig. 2b).

10. Line 208: How many articles were returned by the search. I think it would be good to provide a prisma flow chart for your literature selection http://www.prisma-statement.org/

**Response:** A PRISMA flow chart has been added, along with a more detailed description of the search results in the text and full search results are now provided in the supplementary material.

The text in the methods now reads:

**Lines 278-301:** *“Peer-reviewed articles assessing the effects of ocean acidification on fish behaviour were searched for through Scopus and Google Scholar by J. Clements up until December 21, 2018 using two primary keyword strings: ‘ocean acidification fish behavio(u)r’ and ‘elevated co2 fish behavio(u)r’. The search was conducted using the free software “Publish or Perish” [38] selecting a time period spanning 2009 – 2018, and the maximum number of results that the software allows (1,000 results). Together, the keyword search resulted in a total of 4,411 results, with 2,508 papers remaining for screening after duplicates were removed (Table S3). The titles and abstracts of each article were then screened for initial relevance and inclusion criteria. Articles were included in the database if they included statements of quantitatively assessing the effect of elevated CO_2_ (i.e., ocean acidification) on a behavioural trait of a marine fish; we excluded review articles and papers that measured the effect of elevated CO2 on freshwater fishes and invertebrates. This initial screening resulted in a total of 93 papers being retained from the database search for further evaluation. Five papers were subsequently excluded from the meta-analysis due to a lack of appropriate data for estimating effect size (i.e., variance and/or sample sizes were not provided, or specific behavioural data were not presented), resulting in a total of 88. A cited reference search of the 91 articles was subsequently conducted on March 23, 2019 (just prior to conducting the data analysis) by searching the reference lists and lists of citing articles (on the article’s webpage) and three additional relevant papers were added, for a total of 91 papers included in the meta-analysis. While we did not solicit a call for grey literature, which can be important for meta-analyses [34], such literature online would have been captured in the Google Scholar search; however, no relevant grey literature was uncovered in this search. Final checks of these 91 papers were conducted by both J. Clements and J. Sundin. Results of the literature search are provided in Fig. 5 below. Further details can be found in Table S3, and full search results for each step can be found in the Supplementary File 2.”*

11. Line 218: At first when I read this I found it surprising that you managed to go straight from abstract screening to your full list of 95 analysed papers - usually more fall out when you read the methods and results and realise they haven't done what you thought. Reading further on I realise that they did fall out. I think I would report your sample size as the final number of papers that contributed to the meta-analysis (88 I think). Full prisma flowchart would help with this.

**Response:** A PRISMA flow chart has been added, along with a more detailed description of the search results in the text and full search results are now provided in the supplementary material. See our response to the comment directly above for details.

12. Line 232 - 241: I'm not sure of the value of doing this qualitative analysis. What matters is the actual effect sizes no? Is there reason to think these don't match up? Otherwise its just another way of asking the same question… I think you could more usefully use the space to do some of the other suggested analyses

**Response:** This is a reasonable comment and one that two other reviewers have made. We have thus removed the qualitative analysis from the paper.

13. Line 248-249: Do you mean you just took the effect size for the main effect and ignored interactions, or that you left studies that included other factors out all together? I think most likely the former, but the wording is confusing to me

**Response:** The reviewer is correct that we were trying to relay the former sentiment. We have revised the text here for clarity:

**Lines 327-330:** *“We considered all ocean acidification treatments in our analysis; however, we only included data for independent main effects of ocean acidification, and interactive effects of acidification with other factors (temperature, salinity, pollution, noise, gabazine, etc.) were ignored.”*

14. Line 283: Is it possible to include these by transforming the values so they don't cross 0?

**Response:** We have revised the analysis as per comments from Reviewer 4. Confidence intervals no longer cross 0.

15. Line 366: I'm not sure about this focus on high impact journals here. I wonder if you could just use a funnel plot to show publication bias. I can see why the impact of the journal matters for perception of an effect and citations etc, but for a meta-analysis and estimating an overall effect size it shouldn't matter, and if High profile journals aren't publishing nonsignificant results early on you should still see these results in the literature in low impact journals... it shouldn't really matter. I think trying to get into high impact journals might encourage bad behaviour, but I don't think its high impact journals per se that drives the decline effect.

**Response: As specified in a previous comment above, we can’t really use funnel plots to detect** publication bias due to using effect size magnitudes. However, we have restricted our analysis for “bias” to highlight exactly what the reviewer states: studies with the largest effect sizes tend to have low sample size, but are published in high IF journals and have fuelled citation bias in this field. While the reviewer qualifies this as ‘general publication bias”, we do feel that this is important to highlight quantitatively. Nonetheless, we agree with the reviewer that looking at trends in impact factor and sample size over time does not provide anything more substantial and have removed references to this “driving the decline effect” this from the manuscript. Instead, we simply focus on the fact that there appears to be publication bias that has driven citation bias in this field. This section of the methods has been revised thoroughly to reflect the changes made to the analysis, reflecting that we did not look at the influence of publication bias and sample size on the decline effect *per se*, but visually assess evidence for these biases in our dataset:

**Lines 424-446:** *“****Assessing evidence for selective publication bias, citation bias, and methodological bia****s*

*Alongside testing for the decline effect, we also wanted to determine whether publication bias and/or methodological bias may have contributed to the large effect sizes reported in this field, and whether there was any evidence for citation bias. In new and emerging topics, large effect sizes can be driven by authors and high-impact journals selectively publishing novel and ground-breaking results with large effect sizes [58]. If selective publication bias was evident among studies testing for effects of ocean acidification on fish behaviour, there would be a positive relationship between effect size magnitude and journal impact factor sensu [23]. Thus, to determine if selective publication bias could be present in this field, we visually assessed the relationship between the journal impact factor (for the year of online publication) and the mean effect size magnitude for each study. It is important to note here that we did not compute weighted mean effect size magnitudes for each study, but simply computed the mean of the raw effect size magnitudes as calculated in the section “Visualizing the decline effect using raw effect size magnitudes” above. To check for citation bias, we visually assessed the relationship between impact factor and the number of citations per year (according to Google Scholar on September 10, 2021) for each study, as well as the relationship between mean effect size magnitude and citations per year. If citation bias was present in this field, citations per year would be positively correlated with mean effect size magnitude. Furthermore, if selective publication bias was influencing citation bias, a positive relationship between impact factor and citations per year would be present. Finally, to assess if low sample sizes could contribute to large effect sizes (i.e., higher probability of Type 1 error), we plotted mean effect size magnitude for each study against the mean sample size of that study. If low sample size was influencing effect sizes among studies in this field, large effect sizes would cluster near the lower end of the sample size spectrum.”*

16. I don't think showing a relationship between impact factor and time provides evidence of publication bias - that would occur even if there wasn't publication

**Response:** We agree – see response to the comment above. We agree with the reviewer that looking at trends in impact factor and sample size over time does not provide anything more substantial and have removed references to this “driving the decline effect” from the manuscript. Instead, we simply focus on the fact that there appears to be publication bias that has driven citation bias in this field.

Reviewer #3:

1. Title - "ocean acidification fish ecology" is awkward phrasing.

**Response:** We have altered the title and hope that it is now less awkward:

“*An extreme “decline effect” in ocean acidification effects on fish behaviour*”

2. Line 30 - "Ground-breaking" seems too restrictive. Really, it is the first time that a particular hypothesis or the effect of a certain variable is assessed. The effect size in those first studies is large, and declines thereafter. Whether that work was ground-breaking is not the determinant of this, and the decline effect is not restricted to only truly ground-breaking work.

**Response:** We agree with the reviewer, and what we are referring to is more or less as the reviewer suggests – the first time a hypothesis or effect is published. However, we argue that, by default, the first time a hypothesis or topic is published makes it “ground-breaking” – it is breaking the ground for research on that given hypothesis or effect. This is indeed how we interpret the use of “ground-breaking” here and have elected to keep the text as is, with the addition of a brief definition for “ground-breaking” as suggested by the reviewer:

**Lines 36-38:** *“Publications for new hypotheses or ground-breaking scientific discoveries are often followed by attempts to replicate and build upon the initial research. In many instances, however, follow-up studies fail to replicate initial effects, and/or report smaller effect sizes”*

3. Lines 31 + 32 - it is not really that they fail to replicate the initial effect. It is really that the effect size is smaller. That is a subtle but important distinction.

**Response:** We mostly agree with the reviewer; however, often, follow up studies do indeed fail to replicate initial effects (i.e., find no effect when there previously was an effect). We have amended the text to capture both facets:

**Lines 37-38:** *“In many instances, however, follow-up studies fail to replicate initial effects, and/or report smaller effect sizes”*

4. Line 33 - I am not sure that I can agree that the decline effect itself is what has fueled the reproducibility crisis. It may be one of the drivers, but it is not the only one, and probably not the most important one.

**Response:** This is a good point, and one we agree with. We have removed this sentence to align with the reviewer’s next comment on **Line 38**.

5. Overall, the opening paragraph needs refinement. Alternately, it could be deleted and you can start at line 34.

**Response:** This is a another good suggestion. We have removed the final sentence of this paragraph and combined the first sentence with the second paragraph:

**Lines 36-46:** “*Publications for new hypotheses or ground-breaking scientific discoveries are often followed by attempts to replicate and build upon the initial research. In many instances, however, follow-up studies fail to replicate initial effects, and/or report smaller effect sizes The tendency for initial scientific findings—which can show strong effects with large effect sizes—to lose strength over time is referred to as the ‘decline effect’ [1]. This phenomenon was first described in the 1930s, and has since been documented in a range of scientific disciplines [1], including ecology and evolution [2]. It captures the concept of initial reports with large effect sizes that overestimate reality. In such instances, the early, large effect sizes are the key problem, not the subsequent decline. The decline effect could therefore equally be referred to as the ‘early inflation effect’. Nonetheless, this process can be problematic by delaying accurate scientific understanding of a given phenomenon, and can have applied ramifications, for example, to policy making [3]*”

6. Lines 39-40 - what comes after the semi-colon is unnecessary. Recommend deleting it.

**Response:** The text has been revised as suggested:

**Lines 40-43:** *“This phenomenon was first described in the 1930s, and has since been documented in a range of scientific disciplines [1], including ecology and evolution [2,3]. It captures the concept of initial reports with large effect sizes that overestimate reality.”*

7. Lines 41-42 - should read "using research on THE IMPACT of ocean acidification on fish behaviour" - there are numerous examples through the text where the wording can be improved for clarity.

**Response:** The text has been revised as suggested:

**Lines 65-66:** “*Here, we present a striking example of the decline effect in ecology over the past decade in research on the impact of ocean acidification on fish behaviour.*”

8. Line 44 and elsewhere - tone down the word choice from "drastically". Also, the phrase "appear drastically overestimated" is an phraseology contradiction.

**Response:** This phrasing has been revised here and throughout the manuscript. As an example, the text here now reads:

**Lines 66-70:** “*We find that initial effects of acidification on fish behaviour have all but disappeared over the past five years, and present evidence that common biases influence reported effect sizes in this field. Ways to mitigate these biases and reduce the time it takes to reach a “true” effect size, broadly applicable to any scientific field, are discussed.*”

9. Lines 44 - 45 - do the biases really cause the decline effect?

**Response:** This is a valid point, and one that was raised by Reviewer 2 as well. We have revised the text throughout the manuscript to remove reference to these biases causing the decline effect. Instead, we simply focus on evidence that these biases are present in this field of research. We have added additional data suggesting that citation bias is also present in this field. The text has been amended accordingly here in the Abstract:

**Lines 25-30:** *“The decline effect in this field cannot be explained by three likely biological explanations, including increasing proportions of studies examining (1) cold-water species, (2) non-olfactory associated behaviours, and (3) non-larval life stages. Furthermore, the vast majority of studies with large effect sizes in this field tend to be characterized by low sample sizes, yet are published in high impact journals and have a disproportionate influence on the field in terms of citations.”*

10. Line 45 - "Ways to mitigate the issues…" What ways, and what issues. Either elaborate, or delete.

**Response:** We feel that elaborating on the ways of mitigating biases here in the Introduction is not really necessary – we are simply informing the reader that we will discuss some ways later in the paper. We have amended the text here, however, to specify the “issues”:

**Lines 68-70:** *“Ways to mitigate these biases and reduce the time it takes to reach a “true” effect size, broadly applicable to any scientific field, are discussed.”*

11. Line 47 - "Fishy effects" - I realize that you are using a double entendre, but I advise against it. I would argue that this work will reach, and be accepted by, a wider audience if you refrain from this kind of thing and keep the wording tight and not over-stated or too strong and sweeping.

**Response:** This is a valid criticism, and one that was brought up by another reviewer. We have obliged and removed this subheading from the manuscript.

12. Line 49 - Isn't it a lot more than 300 per year?

**Response:** Perhaps now, but during that time the number of papers hovered just above 300 (as the paper referenced in this sentence states). We have kept the text as is here with modifications according to Reviewer 1:

**Lines 48-50:** *“With more than 300 papers published per year from 2006 to 2015, the exponential growth of ocean acidification studies represents one of the fastest expanding topics in the marine sciences [5], and underscores the perceived risk of ocean acidification to ecosystem resilience”*

13. Line 53 - I am not sure what a "profound" report is. I come back to word choice. Please be more thoughtful about using appropriate words or phrases to explain what you mean.

**Response:** The text has been amended for clarity:

**Lines 53-55:** *“Some of the most striking effects of ocean acidification are those concerning fish behaviour, whereby a series of sentinel papers in 2009 and 2010 published in prestigious journals reported large effects of laboratory-simulated ocean acidification [8–10].*”

14. Line 54 - Another interesting analysis would be to assess whether such journals publish initial strong effect studies more than other journals.

**Response:** We agree that such an analysis would be interesting, but we feel that this falls outside the scope of this paper.

15. Lines 55-58 - Consider - would this be more suitable for a mass media article as opposed to a primary research article?

**Response:** We don’t think so. Underscoring that such studies not only made impact among the general public, but also on policy makers, is certainly warranted in a primary research article, as one goal of science is to influence human effects on nature through policy and public support. We have thus kept the text as written here, though we have removed the anecdote re. “Finding Nemo”:

**Lines 57-59:** *“The severe negative impacts and drastic ecological consequences outlined in those studies were highly publicized in some of the world’s most prominent media outlets [11–13] and were used to influence policy through a presentation at the White House [14].”*

16. Lines 69-70 - the way that this is stated implies that the strength of the effect was determined by the way that the authors interpreted it. I assume that is not the case and that you are really referring to the statistical effect size? Either way, this should be reworded for better clarity-distinction between the qualitative vs. quantitative analysis.

**Response:** The text has been revised for clarity:

**Lines 74-76:** “*Generally, effect sizes in this field have decreased by an order of magnitude over the past decade, from mean effect size magnitudes >5 in 2009-2010 to effect size magnitudes <0.5 after 2015 (Fig. 1a, b; Table S1).*”

17. Line 80 - what is an "outstandingly" large effect size? Please describe how you define it (as opposed to say simply a "large" effect size), or select a more meaningful-accurate word. Perhaps it would just be clearer to say something like the effect sizes of studies after the original ones are almost uniformly smaller?

**Response:** We have removed the term “outstanding” throughout as per comments from other reviewers on toning down the language.

18. Line 85 - elaborate on what you mean by "baseline behaviours".

**Response:** This term has been removed in the revised version of the manuscript. The text has been expanded upon here for clarity according to comments from Reviewer 1:

**Lines 86-106:** *“The large effect size magnitudes from early studies on acidification and fish behaviour are not present in the majority of studies in the last five years (Fig. 1b; Table S1). This decline effect could be explained by a number of factors, including biological. For example, cold-water fish in temperate regions experience a higher degree of temporal variability in carbonate chemistry parameters over large spatial areas [13]. Therefore, they may be less sensitive to changes in seawater CO2 as per the Ocean Variability Hypothesis [14]. As such, if an increasing number of studies on cold-water species over time was responsible for the decline effect, removing cold-water species from the dataset (i.e., only including warm-water species) should result in the decline effect trend disappearing. This was not the case, as the decline effect persisted when only warm-water species were considered (Fig. 2a). In the same vein, the strongest ocean acidification effects on fish behaviour have undoubtedly been reported for chemical cue (herein ‘olfactory’) responses, and increasing numbers of studies on non-olfactory behaviours could explain the decline effect. If this was true, removing non-olfactory behaviours from the dataset should negate the decline effect trend. Again, this was not the case (Fig. 2b). Finally, early studies of ocean acidification and fish behaviour used larval fish, which are typically considered to be more sensitive to environmental perturbations than juveniles and adults. If a greater proportion of studies used less sensitive life stages through time, then removing those life stages and focusing exclusively on larvae should abolish the decline effect. Once again, this was not the case (Fig. 2c). These analyses show that ocean acidification studies on fish behaviour exhibit a decline effect that is not explainable by three biological processes commonly considered important drivers of acidification effects (Fig. 2a-c; Table S1).”*

19. Line 87-88 - they exhibit a decline effect, as opposed to being characterized by one. Reconsider the need to say "strongly" - the reader can see it in the Figure.

**Response:** The text has been amended as suggested, and we elaborated on this decline effect not being explained by three likely biological factors:

**Lines 104-107:** “*These analyses show that ocean acidification studies on fish behaviour exhibit a decline effect that is not explainable by three biological processes commonly considered important drivers of acidification effects (Fig. 2a-c; Table S1).*”

20. Lines 110-111 - selective exclusion of data is very hard to document and assess. You might consider making it clear that you do not have such evidence, you are just making a general comment.

**Response:** The text has been amended as suggested:

**Lines 145-148:** “*While we did not directly test it in our analysis, studies with small sample sizes are also more susceptible to statistical malpractices such as p-hacking and selective exclusion of data that do not conform to a pre-determined experimental outcome, contributing to inflated effects [17].*”

21. Lines 130-135 - If you do not have evidence to document any of this, then make clear that you are speculating, albeit reasonably, about some of the likely drivers of the decline effect.

**Response:** This is a good suggestion, and one raised by another reviewer as well. We have added a statement to the end of this paragraph to make it clear that we did not directly test this empirically:

**Lines 160-171:** *“Experimenter/observation bias during data collection is known to seriously skew results in behavioural research [18]. For example, non-blinded observations are uncommon in life sciences, but are known to result in higher reported effect sizes and more significant p-values than blinded observations [19]. Most publications assessing ocean acidification effects on fish behaviour, including the initial three studies reporting large effect sizes, do not include statements of blinding for behavioural observations. Furthermore, given that statements of blinding can be misleading [20], there has been a call for video evidence in animal behaviour research [21]. Moreover, the persistence of inflated effects beyond initial studies can be perpetuated by confirmation bias, as follow-up studies attempt to confirm initial inflated effects and capitalise on the receptivity of high-profile journals to new (apparent) phenomena [22]. While our analysis does not empirically demonstrate that experimenter bias contributed to the decline effect, it is possible that conscious and unconscious experimenter biases may have contributed to large effect sizes in this field.”*

22. Line 143-147 - not surprising, but good to see it quantified.

**Response:** Thanks - we agree. Note that we have removed the analysis of impact factor over time, as another reviewer pointed out that this does not really show that publication bias drove the decline effect. Nonetheless, we’ve retained the assessment of effect size as a function of impact factor and have added analyses showing that large effect sizes published in these high impact journals have contributed to citation bias in this field.

23. Line 150 - spelling problem here - not sure what "can fue citation bias" means?

**Response:** This should have been ‘fuel citation bias”. The typ has been fixed, and we have added additional data to quantitatively show that the field suffers from citation bias, and that studies today continue to use these early studies to make blanket statements suggesting that OA is predicted to have wide ranging effects on fish behaviour (i.e., see quotes from studies published in 2021  in Table S1):

**Lines 194-199:** “*Together, our results suggest that large effect sizes among studies assessing acidification impacts on fish behaviour generally have low sample size, but tend to be published in high impact journals and are cited more [25]. Consequently, the one-two punch of low sample sizes and the preference of high impact journals to publish large effects has seemingly led to an incorrect interpretation that ocean acidification will result in broad impacts on fish behaviour and thus have wide-ranging ecological consequences – an interpretation that persists in studies published today (Table S2).*”

24. Line 165 - although it is a reasonably high probability, you do not actually provide any evidence of experimenter bias, so I would not overstate that.

**Response:** Reviewer 2 suggested the same. The text has been amended here accordingly:

**Lines 214-223:** *“Our results suggest that large effects of ocean acidification on fish behaviour were at least in part due to methodological factors in early studies (e.g., low sample sizes). Furthermore, the proliferation and persistence of this idea has likely been aided by the selective publication of large effect sizes by authors and journals, particularly at the onset of this field, and the continued high frequency of citations for those papers. It is important to note, however, that low sample size and selective publication cannot fully explain the strong decline effect detected here, and other biases and processes may be at play [5,27]. Nonetheless, we call on journals, journal editors, peer-reviewers, and researchers to take steps to proactively address the issues of low sample size and selective publication, not only in the ocean acidification field, but also more broadly across scientific disciplines.”*

25. Line 166 - what is an "outstanding" effect?

**Response:** We have removed the term “outstanding” throughout as per comets from other reviewers on toning down the language.

26. Line 175 - I am not sure that it would ever be possible to achieve completely unbiased results. Perhaps rephrase, "to limit biases"?

**Response:** The text has been amended as suggested:

**Lines 236-237:** “*Journals, researchers, editors, and reviewers can take additional steps to limit biases in published research.*”

27. Line 177 - pre-registration does not on its own eliminate publication bias.

**Response:** We agree and have revised the text here accordingly. We have also added clarification here that we were referring to registered reports (i.e., journals pre-accepting papers based on sound methodology rather than results and conclusions) as per comments from Reviewer 2:

**Lines 237-244:** *“First and foremost, we suggest that journals adopt the practice of registered reports to ensure that studies not detecting an effect are published in a timely manner. Herein, journals should provide authors with the ability to submit proposed methodologies and have them formally peer reviewed prior to studies even being conducted. If methodologies are deemed sound (or revised to be so) and “accepted” by reviewers, journals should commit to publishing the results regardless of their outcome. While not a silver-bullet solution, this practice could help to minimize selective publication bias and reduce the risk of early, flawed studies being disproportionately influential in a given field [30].”*

28. Lines 186-189 - I find that this is another example of making an unfounded accusation (albeit a likely true one) that will not serve to help your case and which is unnecessary. If you have evidence of it, you can say it. Otherwise, you can just say that editors should use a diversity of reviewers.

**Response:** We actually think it is important to specify why editors should choose diverse reviewers here, as without providing a rationale would make this a somewhat empty suggestion. Nonetheless, we have specified here once again that we do not empirically demonstrate this with our analyses:

**Lines 251-256:** “*When selecting peer-reviewers for manuscripts, editors should also be aware that researchers publishing initial strong effects may be biased in their reviews (i.e., selectively accepting manuscripts that support their earlier publications) and ensure a diverse body of reviewers for any given manuscript. While we do not empirically demonstrate this bias in our analyses, it is important to recognize and mitigate the potential for it to prolong inaccurate scientific findings.*”

29. Line 198-199 - I recommend that you delete this and start with "Our data demonstrate…"

**Response:** The text has been amended as suggested:

**Lines 265-266:** “*Our results demonstrate that more than a decade of ocean acidification research on fish behaviour is characterized by the decline effect.*”

30. Methods. I am not an expert on meta-analysis methodology so I defer to other reviewers who are.

**Response:** Thanks to the reviewer for their transparency. Indeed, Reviewer 4 highlighted some methodological issues in our previous manuscript which we have rectified.

31. Lines 219-200 - the latter part of this sentence is unnecessary.

**Response:** We have removed the text surrounding this being the most comprehensive database to date as suggested.

32. Lines 231-241 -It seems to me that it would have also been interesting to compare the quantitative effect sizes with those that you extracted from the discussion (for the same article)?

**Response:** We have removed the qualitative analysis as per comments from the other 3 reviewers.

Reviewer #4:

This meta-analysis is an exciting and important work showing a decline effect. However, several aspects of methodological procedures and analyses can be much better. I believe that this MS needs to be improved to warrant a place in PLoS Biology, because this meta-analysis (systematic review) will be influential work so that it should have a high standard. Also, it does not cite some previous key papers, which I mention below.

1. It requires better reporting of search results, inclusion/exclusion criteria etc, which are detailed in PRISMA reporting guideline that is used for many systematic reviews. I would at least like to see a PRISMA diagram showing the results of screening processes. One can see an example of this in a very relevant work:

Sánchez-Tójar A, Nakagawa S, Sanchez-Fortun M, Martin DA, Ramani S, Girndt A, Bókony V, Kempenaers B, Liker A, Westneat DF, Burke T. Meta-analysis challenges a textbook example of status signalling and demonstrates publication bias. Elife. 2018 Nov 13;7:e37385.

**Response:** A PRISMA flow chart has been added, along with a more detailed description of the search results in the text and full search results are now provided in the supplementary material.

The text in the methods now reads:

**Lines 278-302:** *“Peer-reviewed articles assessing the effects of ocean acidification on fish behaviour were searched for through Scopus and Google Scholar by J. Clements up until December 21, 2018 using two primary keyword strings: ‘ocean acidification fish behavio(u)r’ and ‘elevated co2 fish behavio(u)r’. The search was conducted using the free software “Publish or Perish” [33] selecting a time period spanning 2009 – 2018, and the maximum number of results that the software allows (1,000 results). Together, the keyword search resulted in a total of 4,411 results, with 2,508 papers remaining for screening after duplicates were removed (Table S3). The titles and abstracts of each article were then screened for initial relevance and inclusion criteria. Articles were included in the database if they included statements of quantitatively assessing the effect of elevated CO_2_ (i.e., ocean acidification) on a behavioural trait of a marine fish; we excluded review articles and papers that measured the effect of elevated CO_2_ on freshwater fishes and invertebrates. This initial screening resulted in a total of 93 papers being retained from the database search for further evaluation. Five papers were subsequently excluded from the meta-analysis due to a lack of appropriate data for estimating effect size (i.e., variance and/or sample sizes were not provided, or specific behavioural data were not presented), resulting in a total of 88. A cited reference search of the 91 articles was subsequently conducted on March 23, 2019 (just prior to conducting the data analysis) by searching the reference lists and lists of citing articles (on the article’s webpage) and three additional relevant papers were added, for a total of 91 papers included in the meta-analysis. While we did not solicit a call for grey literature, which can be important for meta-analyses [34], such literature online would have been captured in the Google Scholar search; however, no relevant grey literature was uncovered in this search. Final checks of these 91 papers were conducted by both J. Clements and J. Sundin. Results of the literature search are provided in Fig. 5 below. Further details can be found in Table S3, and full search results for each step can be found in the Supplementary File 2.”*

2. Sanchez-Tojar et al.'s paper shows the importance of grey literature, which is also mentioned in the PRISMA explanation & elaboration. There is no mention of the grey literature search - the authors did use Google Scholar, which does include grey literature.

**Response:** We did not solicit a call for grey literature as has been done for other evidence synthesis projects, as we wanted to include the most robust reports of OA effects on fish behaviour (i.e., peer reviewed research only). However, the Google Scholar search itself did not reveal any grey literature for this topic. We have added a sentence to the text accordingly:

**Lines 296-299:** “*While we did not solicit a call for grey literature, which can be important for meta-analyses [3], such literature online would have been captured in the Google Scholar search; however, no relevant grey literature was uncovered in this search.”*

3. the authors used absolute values for meta-analysis, and as far as I can see, wrong sampling variances (meta-analytic weights) were used for meta-analysis. Please see this paper:

Morrissey MB. Meta‐analysis of magnitudes, differences and variation in evolutionary parameters. Journal of Evolutionary Biology. 2016 Oct;29(10):1882-904.

AS this paper will show, the current analysis is incorrect. So I recommend using the transform-and-analyze approach.

**Response:** We thank the reviewer for pointing us to this analytical issue with our previous manuscript. With steep learning curve (and some frustration…), we have managed to employ this approach using code adapted from García-Roa et al. (2018). We have also consulted with a leading expert in this analysis to confirm that the present analysis is indeed the best currently developed approach for this type of data. Thus, wherever mean effect size magnitudes (±CI) are presented for a levels of a categorical factor (i.e., year, impact factor bin, and sample size bin), the mean magnitudes and confidence bounds represent those obtained using the approach detailed in García-Roa et al. (2020).

Given this shift in analytical approach, we have removed all reference to “statistical significance” throughout the manuscript, as interpreting significance from effect size magnitudes (i.e., absolute values) is not really appropriate. The relevant section of the methods ow reads:

**Lines 382-399:** *“****Assessing weighted mean effect size magnitudes by year.*** *Although useful for visualizing a trend in effect sizes over time, the first approach above is not analytically rigorous. Properly analyzing trends in effect sizes should including a weighted component whereby individual effect sizes are weighted according to their precision (i.e., measurements with a larger sample size and lower variance should be given more weight than those with a lower sample size and higher variance) [57]. As such, we computed weighted mean effect size magnitudes (and their associated uncertainty; i.e., upper and lower confidence bounds) for each year represented in our dataset, and assessed the trend in these effect sizes over time.*

*Weighted mean effect size magnitudes (lnRR) and their confidence bounds were computed using the “transform-then-analyze” approach as suggested by [58], with R code adapted from [59] to avoid biased estimates of effect size magnitude. Briefly, this method estimates the mean effect size for each level of a moderator of interest (i.e., each year in our dataset) by assuming a normal distribution and subsequently transforming the mean effect size using a folded normal distribution to estimate a mean effect size magnitude. Uncertainty around the mean effect size magnitude was estimated in a Bayesian fashion using the MCMCglmm() function from the MCMCglmm package [60], applying the entire posterior distribution of mean estimated to the folded normal distribution as per [59]. Annotated R code and all raw data files for the analysis are provided in the supplementary material for replicability (Supplementary files 4–11).”*

In addition to the revised analysis, we have included a second visualization for the decline effect, which shows the average absolute effect size for each experiment (i.e., raw data) plotted over time and fitted with a loess curve (see Fig. 1a).

**Reference:**

García‐Roa R et al. (2020) Biol. Rev. 95: 1607–1629.

4. the authors dichotomized continuous variables (e.g., strong effect, N > 30 etc). I strongly advise against such an approach (leading to Type I errors). Please see this paper:

Royston, Patrick, Douglas G. Altman, and Willi Sauerbrei. "Dichotomizing continuous predictors in multiple regression: a bad idea." Statistics in medicine 25.1 (2006): 127-141.

**Response:** This was also suggested by two other reviewers, and we have obliged. The qualitative analysis has been removed from the paper.

5. I would recommend using the better version of lnRR (small sample size corrected one) proposed by this paper:

Lajeunesse MJ. Bias and correction for the log response ratio in ecological meta‐analysis. Ecology. 2015 Aug;96(8):2056-63.

**Response:** A good suggestion. However, we have instead used a Bayesian approach to estimate mean effect size magnitudes and their variance using the approach detailed in García-Roa et al (2020).

**Reference:**

García‐Roa R et al. (2020) Biol. Rev. 95: 1607–1629.

6. There are several key relevant papers missing. For example:

Jennions MD, Møller AP. Relationships fade with time: a meta-analysis of temporal trends in publication in ecology and evolution. Proceedings of the Royal Society of London. Series B: Biological Sciences. 2002 Jan 7;269(1486):43-8.

Koricheva, Julia, and Elena Kulinskaya. "Temporal instability of evidence base: a threat to policy making?." Trends in ecology & evolution 34.10 (2019): 895-902.

Murtaugh PA. Journal quality, effect size, and publication bias in meta‐analysis0000-00-00 0:00:00 AM. Ecology. 2002 Apr;83(4):1162-6.

**Response:** Thanks to the reviewer for pointing us to these papers. All three have now been referenced appropriately in the text:

1) Jenions & Møller (reference [2]):

**Lines 40-42:** “*This phenomenon was first described in the 1930s, and has since been documented in a range of scientific disciplines [1], including ecology and evolution [2,3].”*

**Lines 174-176:** “*Indeed, publication bias has been suggested as perhaps the most parsimonious explanation for the decline effect in ecology and evolution, as studies showing no effect can be difficult to publish [2].*”

2) Koricheva & Kulinskava (reference [4]):

**Lines 45-46:** “*Nonetheless, this process can be problematic by delaying accurate scientific understanding of a given phenomenon, and can have applied ramifications, for example, to policy making [4].*”

3) Murtaugh (reference [27]):

**Lines 192-193:** “*Similar results have been reported in other areas of ecology and evolution, perhaps most notably in studies regarding terrestrial plant responses to high CO2 [27].*”

**Lines 429-431:** “*If selective publication bias was evident among studies testing for effects of ocean acidification on fish behaviour, there would be a positive relationship between effect size magnitude and journal impact factor sensu [27].”*
