# Supplementary material for: Meta-analysis reveals an extreme “decline effect” in the impacts of ocean acidification on fish behavior
Source: PLoS Biol. 2022 Feb 3;20(2):e3001511. doi: 10.1371/journal.pbio.3001511 (PMC8812914; doi:10.1371/journal.pbio.3001511)
Supplement: Attachment — Submitted filename: R2 - Response to editors and reviewers.docx [file pbio.3001511.s019.docx]

**Response to editors and reviewers**

**Editor comments**

Thank you for submitting a revised version of your manuscript "An extreme “decline effect” in ocean acidification effects on fish behaviour" for consideration as a Meta-Research Article at PLOS Biology. This revised version of your manuscript has been evaluated by the PLOS Biology editors, the Academic Editor and the original reviewers.

In light of the reviews (below), we are pleased to offer you the opportunity to address the remaining points from the reviewers and the Academic Editor in a revised version that we anticipate should not take you very long. We will then assess your revised manuscript and your response to the reviewers' comments and we may consult the reviewers again.

**Response:** We want to thank the editors and reviewers for a fast and thorough second round of peer review on our manuscript. We are delighted that all parties seem pleased with our revised manuscript. We respond to the remaining comments from the editors and reviewers below.

a) Please attend to the remaining requests from reviewer #1.

**Response:** See responses to Reviewer 1’s additional comments below.

b) We are aware that some of you harbour suspicions about the scientific validity of two key studies that are analysed here (e.g. https://www.science.org/content/article/does-ocean-acidification-alter-fish-behavior-fraud-allegations-create-sea-doubt). Indeed, that Science article cites the preprint of the current submission. Given this, the Academic Editor has made the following request: "The authors should do a formal re-analysis without those two studies. I don't really get how this is a decline effect if it is because of misconduct rather than poorly designed experiments or simply a regression to the mean effect. In other words, is "misconduct" a plausible mechanism for the decline effect, or does it have to be something else? In which case, the leverage of those two studies on the meta-analysis seems particularly important."

**Response:** This is correct. While the results of formal investigations are not yet available, we have added the additional analysis according to the editor. In short, we have added to the manuscript an additional visualization akin to Fig. 1 excluding the studies authored or co-authored by the lead investigators from the initial studies in 2009 and 2010 (now Fig. 5 in the manuscript):

We first ran the analysis as suggested by the editor by excluding only the initial studies in 2009-2010. There was still a decline effect that was apparent in the dataset (albeit less striking), with studies published from 2011-2014 exhibiting a higher effect size than those published from 2015 onward (note that this can also be seen in Fig 1b by looking at mean effect size magnitudes after 2011):

**The decline effect in ocean acidification research on fish behaviour excluding initial studies.** Trend in raw effect sizes (lnRR) for each experiment in our dataset excluding the three initial studies published in 2009–2010, plotted as a function of year of publication (online) and colour-coded according to study. Data are fit with a Loess curve with 95% confidence bounds. Source data for each figure panel can be found in Supplementary File 14.

However, it is important to note that many other studies led by these authors are also under formal investigation (as per the *Science* article mentioned in the editor’s comment). To address this, we have added additional formal analyses excluding all studies authored or co-authored by the lead investigators of those initial studies (i.e., P. Munday and/or D. Dixson). Based on this analysis, the decline effect is no longer apparent when these authors are removed from the dataset (note that there are no publications not co-authored by these investigators, and thus no effect size data, prior to 2012 for this analysis):

**Fig. 5. The decline effect in ocean acidification research on fish behaviour excluding studies authored (or co-authored) by lead investigators of initial studies. (a)** Trend in raw effect sizes (lnRR) for each experiment in our dataset excluding all studies authored (or co-authored) by lead investigators of the three initial studies plotted as a function of year of publication (online) and colour-coded according to study. Data are fit with a Loess curve with 95% confidence bounds. **(b)** Mean effect size magnitude (absolute lnRR magnitude ± upper and lower confidence bounds) for each year of publication (online) in our dataset excluding all studies authored (or co-authored) by lead investigators of the three initial studies. Mean effect size magnitudes and confidence bounds were estimated using Bayesian simulations and a folded normal distribution. Note: colours in (b) are aesthetic in nature and follow a gradient according to year of publication. Also note that data begin in 2012 since all publications prior to this year included initial lead investigators in the author list. Source data for each figure panel can be found in Supplementary File 13.

We have elected to add the latter analysis to the manuscript as per the editor’s request. Given the addition of these new results, we have added a cautious section to the text to incorporate them into the manuscript:

**Lines 246-259:** *“****Investigator effects.*** *It is important to note that the early studies published in 2009-2010 [8–10], and some subsequent papers from the same authors, have recently been questioned for their scientific validity [30]. Indeed, these early studies have a large influence on the observed decline effect in our analysis. It is thus important to explore the potential for investigator effects, as such effects have been reported to drive decline effects for the field of ecology and evolution in the past (e.g., fluctuating asymmetry [31]). When all papers authored or co-authored by at least one of the lead investigators of those early studies were removed from the dataset (n = 41 studies, 45%), the decline effect was no longer apparent from 2012–2019 (Fig. 5). While conclusions regarding the potential roles of invalid data await further investigation [30], our results do suggest that investigator or lab group effects have contributed to the decline effect reported here. We suggest that future studies documenting the presence or absence of decline effects – and indeed meta-analyses in general – should carefully consider and evaluate whether investigator effects may be at play in a given field of study.”*

We have also added an appropriate explanation of the new analyses to the Materials and Methods section (in the section which is now subtitled “**Assessing evidence for selective publication bias, citation bias, and methodological bias, and investigator effects**”):

**Lines 538-547:** *“Finally, because the validity of data presented in the early studies of this field have recently been questioned [30] and investigator bias has been reported to drive decline effects in ecology and evolution in the past [31], we tested for investigator (or lab group) effects by re-running the analysis on a dataset with all papers authored or co-authored by the lead investigators of those initial papers (i.e., P. Munday and/or D. Dixson) removed. Herein, we once again visualized all raw effect sizes plotted against time (i.e., see Visualizing the decline effect using raw effect size magnitudes), and also computed weighted mean effect size magnitudes for each year (i.e., see Assessing weighted mean effect size magnitudes by year). The potential for investigator effects influencing the decline effect would be apparent if the decline effect was not evident in the dataset excluding these authors.”*

The code for this new analysis is also contained I the updated Supplementary File 3, the raw data for the analysis has been added as Supplementary file 12, and the source data for the figures has been added to the new Supplementary File 13.

c) Please address my Data Policy requests below; specifically, please supply numerical values underlying Figs 1ABCD, 2ABC, 3, 4ABCDE, and cite the location of the data clearly in each relevant Fig legend (I note that the raw data and code are already provided, but we’ll need the above output values too). These can be included as supplementary data files (S1_Data.xlsx, etc.) or in a public repository such as Dryad, Github, Figshare, etc.

**Response:** All source data for each figure panel are now provided in Supplementary File 15. Each figure caption now refers to this Supplementary File accordingly, with text reading:

*“Source data for each figure panel can be found in Supplementary File 13.”*

**Reviewer 1 comments**

I would like to thank the authors for seriously considering all of the reviewer comments. I found this manuscript to be greatly improved from the prior version. I must also say that the authors convinced me that one of my concerns was clearly misplaced. Specifically, the premise that a few early papers had an outsized impact on the observed effect, and that these papers were no longer held up as representative of the field. I was very surprised to see that these papers are still receiving 60-80 citations a year! I have a few minor suggestions for the authors to consider, but otherwise I think the authors have done a nice job revising this work.

**Response:** We want to sincerely thank the reviewer for their time and effort with our manuscript – the paper is certainly much better and more comprehensive as a result of this reviewer’s comments. We respond to each of the additional suggestions below. Thanks again!

#1) The references to citations (beginning on line 201 and Figure 4) are very useful, but Google Scholar was a curious choice. Considering this is being used to compare to impact factor, I think that ISI citations are a much better and more accurate measure of the peer reviewed literature.

**Response:** While we see the merit in associating ISI citations to journal impact, it is important to consider that high IF papers can also garner more citations outside of the traditional literature, as they also garner more media attention and, by default, have a broader outreach. As such, we feel that using a citation tracking database that captures the most citations is a more robust approach. To this end, Google Scholar wins the race against ISI sources. Across a broad range of fields, Google Scholar not only captures the vast majority of citations caught by other sources (including ISI sources and Scopus), but also captures substantially more citations that are missed by ISI and Scopus (Harzing & van der Wal 2008; Martín-Martín et al. 2018). As such, we contend that Google Scholar is the superior source for citation data and have elected to keep the citation data as they were. We have added text to the Materials and Methods justifying the use of Google Scholar as above:

**Lines 525-529:** *“We chose to use Google Scholar for citation data because it has been shown to be more comprehensive than other sources (e.g., Web of Science, Journal Citation Reports, and Scopus), as Google Scholar not only captures the vast majority of citations documented by these other sources, but tends to capture more citations that are missed by those sources [65,66].”*

**References:**

Harzing A, van der Wal R. (2008) Google Scholar as a new source for citation analysis. Ethics Sci Environ Polit. 8: 61–73. doi:10.3354/esep00076

Martín-Martín A, Orduna-Malea E, Thelwall M, Delgado López-Cózar E. (2018) Google Scholar, Web of Science, and Scopus: A systematic comparison of citations in 252 subject categories. J Inform. 12: 1160–1177. doi:10.1016/j.joi.2018.09.002

#2) Line 259-268: Recent events, which I will not detail here, have put the ocean acidification decline effect in a different light. I agree with the authors points about scrutinizing methods, best practices, data transparency and general skepticism, but I wonder if the publishing suggestions should be reconsidered. I don't think registered reports or pre-acceptance of approved methods would have had any positive impact on this particular situation. To me, the authors hit it on the head with the skepticism comment. I would only add that the scientific community was also slow to absorb the published negative results, which are clearly cited less than the initial studies. I would hope this is not the fault of people directly in the area, who are up to date on the newest work. My guess is many of these citations come from ancillary fields that cite high impact work in broad terms. I don't have a solution to that problem, but it seems that it is definitely more the fault of scientists not being thorough in their literature research than it is the fault of the publishing system.

**Response:** The first part of this comment is also something that was flagged by the Academic Editor, who requested some additional analyses to address this point. Based on that editor’s requests, we have added analyses using datasets excluding the lead authors of those initial studies (i.e., P. Munday and/or D. Dixson) to assess whether or not authors (or lab groups) may have influenced the decline effect, and results indeed suggest such (see new Fig. 5 and response to Editor Comment **b** above).

Given this, we agree with the reviewer that registered reports my not have been sufficient to avoid such investigator effects; however, they may reduce the amount of time that inflated results drive an improper understanding of given phenomena. We feel it important to highlight registered reports, because we do think that questionable scientific validity driving decline effects is likely an exception to the norm. As such, we have elected to keep the text regarding registered reports with the caveat that this may not be sufficient to avoid issues such as poor or invalid data:

**Lines 298-308:** *“First and foremost, we suggest that journals adopt the practice of registered reports to ensure that studies not detecting an effect are published in a timely manner. Herein, journals should provide authors with the ability to submit proposed methodologies and have them formally peer reviewed prior to studies even being conducted. If methodologies are deemed sound (or revised to be so) and “accepted” by reviewers, journals should commit to publishing the results regardless of their outcome. Although registered reports may not be sufficient to avoid the influence of some issues such as poor data, they may reduce the risk of inflated results driving decline effects – and prolonged incorrect understanding – for other phenomena in the future. While not a silver-bullet solution, this practice could help to reduce selective publication bias and the risk of early, flawed studies being disproportionately influential in a given field [32].*

In addition, we have added the reviewer’s suggestion that researchers have been slow to pick up on the negative/null result train (this is a nice suggestion – thanks!):

**Lines 327-330:** *“Nonetheless, the citation analysis provided herein suggests that researchers have been slow to adopt studies reporting negative and null results for this field, as the early studies with large effect sizes remain the most highly cited among all articles in our dataset.”*

As for fault, we would argue that it is likely a combination of researchers not being thorough *and* how the publishing system is perceived. High IF journals are often perceived as journals that publish studies of the highest scientific rigor, and results published in them are thus trusted more by a large number of researchers – yet we know this is not the case. We have not added any text to the paper to this end, but these are certainly ideas that are worth pondering and perhaps exploring empirically in the future.

**Reviewer 4 comments**

The authors have very carefully addressed my comments and also the comments of other reviewers. It will be a very important contribution!

**Response:** thanks to the reviewer for their time and effort with our manuscript. We are pleased to have adequately addressed all of their comments.
