# Supplementary material for: Meta-analysis reveals an extreme “decline effect” in the impacts of ocean acidification on fish behavior
Source: PLoS Biol. 2022 Feb 3;20(2):e3001511. doi: 10.1371/journal.pbio.3001511 (PMC8812914; doi:10.1371/journal.pbio.3001511)
Supplement: Attachment — Submitted filename: R3 - Response to Editors.docx [file pbio.3001511.s020.docx]

Response to editors

**a)** We're struggling with the Title a bit. One problem is that "decline effect" is not a widely understood phrase; the second is the duplication of the word "effect," and the third is the absence of the method used; overall the title is currently quite opaque. We suggest something like "Systematic review and meta-analysis suggests that ocean acidification has a negligible direct impact on fish behaviour," if you feel happy with that; however, I'm open to discussing alternatives.

**Response:** We agree with the need for stating the method being used and the duplicate use of the term effect. However, we would like to retain the use of “decline effect”. While negligible effects appear to be the likely “true” effect of OA on fish behaviour, the main finding and story of the paper is centered on the decline effect and biases that have influenced it in this field; thus, we feel that it is important to retain this term in the title. We also question the need to include both “systematic review” and “meta-analysis” in the title, as it seems a little redundant and makes the title quite long-winded and clunky. We list three options in order of our preference:

*Meta-analysis reveals an extreme “decline effect” in the impacts of ocean acidification on fish behaviour*

*Systematic review and meta-analysis reveal an extreme “decline effect” in the impacts of ocean acidification on fish behaviour*

*Impacts of ocean acidification on fish behaviour show an extreme “decline effect”: a systematic review and meta-analysis*

We have included the second option in all accompanying documents to fit most closely with the editors’ suggestion, but we are available to discuss options with the editors as required.

Furthermore, as the editors noted, the term “decline effect” may not be widely understood at first glance. To facilitate the use of this term in the title, we now provide an adequate definition of this term up front in the Abstract:

Lines 27-29: *“Indeed, the phenomenon of decreasing effect sizes over time is not uncommon and is typically referred to as the “decline effect”.”*

**b)** After some debate, and partly because of the specific relevance to this field, we think that it would be more appropriate to publish your paper as a regular Research Article, rather than a Meta-Research Article. This has no formatting implications, but please can you change the Article Type to "Research Article" when you resubmit?

**Response:** No problem – we are quite fine with this. The manuscript has been resubmitted as a Research Article.

**c)** As this is a systematic review and meta-analysis, do check that you've complied with our policy by providing a completed PRISMA checklist and flow diagram. I see that Fig 6 is a flow diagram, but please check our policy here:

<https://journals.plos.org/plosbiology/s/best-practices-in-research-reporting#loc-reporting-guidelines-for-specific-study-types>

**Response:** We now provide a complete PRISMA checklist in the Supplementary Material (new Supplementary File 14) alongside the flowchart in Figure 6, which we have also updated to comply with current PRISMA standards. We have added text to the Methods section to specify these additions (with the appropriate reference for PRISMA 2020 guidelines):

Lines 352-354: *“A systematic literature search was conducted according to PRISMA guidelines [40]; a completed PRISMA checklist can be found in Supplementary File 14 and a flowchart is provided below (Fig. 6).”*

**d)** Please address my Data Policy requests below; specifically, please supply numerical values underlying Figs 1ABCD, 2ABC, 3, 4ABCDE, and cite the location of the data clearly in each relevant Fig legend (I note that the raw data and code are already provided, but we’ll need the above output values too).

**Response:** In the previous resubmission, we had supplied the numerical values underlying the points and error bars for each figure panel in an additional supplementary file (Supplementary File 14) and cited the location of these data in each of the figure legends. As far as we understand, this is still what is being requested here, but perhaps we are misunderstanding something. As previous, we provide the specific raw values for each data point and any associated error bars for each of the figure panels in Supplementary File 14 and refer the reader to Supplementary File 14 in each figure caption. If something more detailed is required, some extra guidance and detail on exactly what is need would be much appreciated.

**e)** Because of a slight nervousness about the possible reaction of Munday, Dixson and co, I've been asked to refer this paper up the management chain, just to check that we don't need legal counsel. I personally think that you've worded everything in a scholarly and appropriately cautious manner; for anything stronger, the reader is merely referred to Enserink's Science article. However, I just thought I'd give you a heads-up just in case the powers-that-be come back and want something tempering.

**Response:** This is understandable, and we very much appreciate the transparency here! We have tried to word the text in a cautious and scholarly manner, but are happy to oblige with any further revisions to this text that may be required.

As you address these items, please take this last chance to review your reference list to ensure that it is complete and correct. If you have cited papers that have been retracted, please include the rationale for doing so in the manuscript text, or remove these references and replace them with relevant current references. Any changes to the reference list should be mentioned in the cover letter that accompanies your revised manuscript.

**Response:** We have double checked all references for formatting and to ensure that all citations in the reference list are contained in the text. One reference was added during this final revision to properly cite the PRISMA guideline statement (Page et al. 2021).
